# Global Sentiment Toward Health AI at the Dawn of the ChatGPT Era: Empirical Analysis of Twitter (X) Discourse

**DOI:** 10.2196/80346

**Published:** 2026-05-05

**Authors:** Lily Minh Wass, Zhengdong Wu, José Vizoso, Joseph T Wu, Leesa Lin

**Affiliations:** 1 Laboratory of Data Discovery for Health Hong Kong China (Hong Kong); 2 WHO Collaborating Centre for Infectious Disease Epidemiology and Control School of Public Health, Li Ka Shing Faculty of Medicine University of Hong Kong Hong Kong China (Hong Kong); 3 Moonrise Initiative Hong Kong China (Hong Kong); 4 The University of Hong Kong – Shenzhen Hospital Shenzhen China; 5 The Hong Kong Jockey Club Global Health Institute Hong Kong China (Hong Kong); 6 Department of Infectious Disease Epidemiology and Dynamics London School of Hygiene & Tropical Medicine London, England United Kingdom

**Keywords:** AI, artificial intelligence, health care, social listening, sentiment analysis, ChatGPT, trust

## Abstract

**Background:**

Artificial intelligence (AI) is increasingly proposed for use in health and health care systems. Beyond technical performance, public perceptions and affective responses influence whether AI technologies are accepted and adopted in real-world contexts. Social media platforms such as X (formerly Twitter) provide large-scale, real-time insight into public discourse surrounding emerging technologies, yet remain underused for examining how health AI is discussed, evaluated, and emotionally framed.

**Objective:**

This study aimed to develop and apply large language model (LLM)–based methods for exploratory social listening on health AI. This is the first study to map large-scale sentiment, emotional expressions, and confidence-related signals in online discussions of applications of AI to health.

**Methods:**

We collected 786,750 English-language posts from X (Twitter) published between January 1 and December 5, 2023, using health- and AI-related keywords. We benchmarked an LLM-based annotation framework by using OpenAI’s GPT-3.5-Turbo and GPT-4, comparing model classifications with trained human researchers. Annotations included overall sentiment and 6 evaluative domains frequently referenced in the literature surrounding attitudes toward health AI—usefulness, safety, privacy, ethics, quality, and trust. After cleaning, GPT-3.5-Turbo used the best-performing prompts to label 388,009 posts. A subset (n=268,347) was further analyzed using Emollama-7b, an open-source model fine-tuned from Meta’s LLaMA2-7B, for emotion detection, and latent Dirichlet allocation for thematic analysis. Comparisons were made across World Health Organization regions.

**Results:**

Compared against human annotations, optimized prompts achieved weighted *F*_1_-scores above 0.60 across evaluative domains and sentiment classification. Global discourse about health AI was 65.26% (95% CI 65.11%-65.4%) positive and 83.62% (95% CI 83.48%-83.76%) emotionally optimistic, although substantial regional variation was observed in sentiment (*P*<.001). The Eastern Mediterranean and South-East Asia regions expressed significantly higher levels of positive sentiment and evaluative agreement in the studied features of health AI, alongside frequent discussion of the tech industry and commercial development. In comparison, the Western Pacific region expressed lower confidence and significantly more mentions of research topics (19.27%, 95% CI 18.5%-20.07%). Privacy was the most prominent global concern, with 33.31% (95% CI 32.98%-33.66%) of privacy-related posts expressing perceived risks. In the Region of the Americas, 18.19% (95% CI 17.92%-18.44%) of posts discussed algorithms and data governance, significantly higher than overall.

**Conclusions:**

This study offers the first systematic characterization of online health AI discourse at scale, mapping stances toward key features of AI, emotional tone, and discussion topics across regions. LLM-powered social listening is demonstrated as a feasible approach for identifying dominant narratives and regionally distinct concerns, capable of surfacing opinions absent from traditional media. This can extend to studying discourse on other evolving health technologies where public surveying is limited. While methodological refinement and multilingual expansion are needed, this framework can inform timely policy development, risk communication, and responsible health AI governance.

## Introduction

The release of large language model (LLM) chatbots like ChatGPT (OpenAI) in 2022 marked a turning point in the accessibility of artificial intelligence (AI), sparking public and research interest alike. By 2023, the rapid progress of LLMs extended to health care applications, leveraging the explosion of online data for training sophisticated AI models [1]. Although thousands of studies on AI in health care are published annually [2], most remain at the proof-of-concept or pilot stage. Progress toward routine clinical deployment has been comparatively slow, reflecting unresolved challenges in validation, generalizability, regulatory approval, workflow integration, and safe implementation in high-stakes clinical environments [3,4].

As with vaccination and other system-level medical interventions, the impact of health AI depends not only on technical performance but on the stability of the social systems that maintain its use. In this sense, public and professional confidence functions as a form of infrastructure—less visible than algorithms or regulatory approvals, yet equally foundational to safe and scalable implementation. Without durable trust in safety, fairness, accountability, and data governance, even high-performing AI tools may fail to achieve uptake.

Although AI’s empirical capabilities are advancing rapidly, its translation into routine care is mediated by perception, politics, and institutional legitimacy [5,6]. Evidence shows that clinicians and the public hold complex, sometimes ambivalent views of health AI, shaped by concerns about safety, bias, accountability, ethics, and data use [6-9]. In high-stakes clinical settings, where consequences are immediate and personal, perceived risks can outweigh demonstrated benefits. Recognizing confidence as infrastructure implies that it must be actively maintained. Scholars have therefore called for continuous, scalable systems to monitor public and professional confidence in health AI over time—analogous to vaccine-confidence surveillance—so that governance, communication, and implementation strategies can adapt to emerging risks and concerns [10].

One promising source of data for examining evolving perceptions of health AI is online platforms, where AI-related discussions surged following the release of ChatGPT in late 2022 [11]. Social media has surpassed traditional media as a major source of information, exerting considerable influence on public opinion formation and politics [12]. Platforms provide sites for exchange and exposure to new ideas in a public forum, yet not all ideas are amplified equally. Visibility on public forums is shaped by engagement-based algorithms, which can tend to reward emotionally charged content.

A growing body of research indicates that content that invokes negative sentiments or high-arousal emotions, such as anger and sadness, is more likely to be perceived as believable and to spread widely [13,14]. This emotional content has boosted the dissemination of misinformation and fake news [15,16]. Because public awareness of health AI is generally low, even among some medical professionals [7,8,17], information consumers might rely on their emotional responses over reasoning when consuming health AI-related media, a choice associated with greater susceptibility to misinformation [18].

Measuring attitudes toward AI is itself a conceptual challenge. Unlike many previous technological innovations, AI systems exhibit anthropomorphic traits, broad applications, and inaccessible “black box” decision-making processes [19,20] that users struggle to conceptualize. Consequently, theoretical approaches to understanding confidence in AI span multiple domains, including technical performance, ethical governance, legal accountability, and societal alignment, without a clear consensus on priorities [20,21]. Support for one attribute of a health AI system, such as predictive accuracy, does not necessarily imply endorsement of others, such as ethical data use or privacy protections [21]. Therefore, a large-scale investigation into the cues in health AI confidence and emotion shared in online discourse is needed to better understand the information environment shaping public perceptions and governance challenges.

Achieving such large-scale insight presents additional methodological obstacles. Previous studies examining social media discourse on health AI have used manual content analysis, restricting analyses to a few thousand posts [10,22]. To address scalability, machine learning approaches like support vector machines have also been explored, but these models often exhibit poor generalizability, particularly when applied to health-related social media data [23,24]. Common natural language processing (NLP) techniques struggle with domain-specific language, sarcasm, and context-dependent framing, and require large, manually annotated datasets and extensive tuning to achieve acceptable performance [24,25]. These constraints in methodology and performance limit the feasibility of timely and large-scale assessments of public opinion.

LLMs have been proposed as a more adaptive and scalable alternative. By leveraging transformer-based architectures, LLMs can capture complex linguistic nuances and adapt to different contexts, while reducing reliance on task-specific training data [26]. Existing research has found LLMs to outperform traditional methods with significantly less reliance on manually annotated data, further reducing the time and resource burden of traditional approaches [27,28]. However, empirical applications of LLMs to the analysis of public discourse on health AI remain limited, while reported performance on health-related social media data has been inconsistent [29,30].

In response to these gaps, this study has 2 primary objectives. First, we evaluate the performance of 2 widely used GPT-based LLMs for annotating short, health AI–related social media posts, using zero-shot, few-shot, and chain-of-thought prompting. This analysis offers a reference point that can be revisited as language models and prompting strategies continue to evolve. Second, drawing on the best-performing annotation approach, we conduct a large-scale, descriptive social listening analysis to characterize how health AI is discussed online. Specifically, we examine the distribution of sentiments, discrete emotions, and thematic content expressed in posts related to health AI, and assess variation across geographic sources of posts. Together, these contributions demonstrate the feasibility and limitations of using LLMs for scalable analysis of public discourse on a rapidly evolving topic.

## Methods

### Overview

A multipronged approach of LLM and NLP tools was used to assess the global sample of X (formerly Twitter) discussions regarding health AI. Post sentiment and positions on health AI confidence were analyzed using an LLM-prompting strategy. Performance was measured using a benchmark dataset and a prompt testing pipeline. Previously validated LLM and NLP methods were applied for emotion detection and discussion themes [31,32].

### Sentiment Analysis Framework

We grounded our exploratory social-listening approach to health AI in methodological advances from vaccine-confidence surveillance, adapting validated, double-coded annotation frameworks that have been applied to large-scale social media data over the past decade. This approach leverages established protocols for construct definition, coder adjudication, and reliability benchmarking to enable systematic monitoring of evolving public attitudes [33-35]. These studies have adapted the World Health Organization’s (WHO) Confidence, Complacency, and Convenience (“3 Cs”) model of vaccine hesitancy [36] to translate a complex decision-making model into analyzable indicators of online discourse (eg, expressions that vaccines are not safe). With this precedent, we reviewed existing studies and the models used to measure attitudes toward AI, such as the prominent technology acceptance model [37] and its extensions. From the literature, we identified 6 features of health AI recurrently discussed by experts and the public (Table 1). Based on their frequency in the literature, we expected these dimensions to be similarly reflected in an exploratory, high-level analysis of how health AI is discussed online.

**Table 1:** Annotation categories for posts about health artificial intelligence on X from January to December 2023. Performance measured using GPT-3.5-Turbo and GPT-4 models in September 2024.

AI^a^ confidence	Definition	GPT-3.5-Turbo performance, weighted *F*_1_-score (95% CI)	GPT-4 performance, weighted *F*_1_-score (95% CI)
Sentiment	The post is positive, negative, or neutral toward AI	0.71 (0.62-0.79)	0.74 (0.67-0.81)
Safety [17,21,38]	This post indicates AI is safe	0.64 (0.56-0.72)	0.79 (0.72-0.86)
Usefulness [6,7,38-41]	This post indicates AI is useful	0.69 (0.60-0.76)	0.77 (0.69-0.84)
Trustworthiness [7,17,37]	This post indicates AI is trustworthy	0.61 (0.52-0.70)	0.63 (0.56-0.71)
Privacy [6-8,17,37,38,40]	This post indicates AI respects privacy	0.66 (0.57-0.74)	0.76 (0.68-0.82)
Ethics [8,17,38-40]	This post indicates AI is ethical	0.63 (0.55-0.71)	0.71 (0.62-0.78)
Quality [6,7,17,38,40]	This post indicates AI is of good quality	0.70 (0.61-0.78)	0.76 (0.67-0.83)

^a^AI: artificial intelligence.

### LLM Performance Validation

We implemented an LLM-based sentiment analysis and validation pipeline to assess model performance in processing health AI-related text, aiming to improve communication strategies in health care. Using the operational definitions in Table 1, researchers manually labeled a benchmark dataset of 150 posts per health AI domain and overall sentiment variable. Benchmark posts were randomly selected from a separate set of health AI-related posts shared outside of the study period. Posts were labeled as “true,” “false,” or “irrelevant” for their stance within each domain (eg, True: Health AI is safe, False: Health AI is not useful, and Irrelevant: Ethics of Health AI is not mentioned) and as “positive,” “negative,” or “neutral” for overall sentiment. In total, 50 posts were labeled per class.

The benchmark size was selected to support comparative evaluation of model performance under realistic annotation constraints, in line with previous evaluations of LLMs for short text annotations [42-44]. Additional concepts of “importance” and “accessibility” were initially included as dimensions of confidence, but due to scarcity in the X sample of “false” labeled posts (ie, Health AI is not accessible and Health AI is not important), these domains were excluded. This resulted in 1050 total annotations for 6 domains and overall sentiment.

The performance of LLMs GPT-3.5 Turbo (OpenAI) and GPT-4 (OpenAI) was assessed using 4 prompt types—zero-shot, few-shot [45], zero-shot chain-of-thought (CoT), and few-shot CoT [46]. Prompts instructed GPT to classify benchmark posts based on their expressed opinion on the 6 health AI domains and their overall sentiment toward the technology according to the format in Table 2.

**Table 2:** Template of zero-shot, few-shot, zero-shot chain of thought, and few-shot chain of thought prompts passed to GPT-3.5-Turbo and GPT-4 to annotate benchmark dataset. Adjective refers to health artificial intelligence concepts defined in Table 1.

Prompt type	Format
Zero-shot	Classify tweets as being in agreement (true), disagreement (false), or irrelevant with the belief that AI^a^ is [adjective].
Few-shot	Tweet: Example Tweet 1 Answer: True/False/Irrelevant Tweet: Example Tweet 2 Answer: True/False/Irrelevant Tweet: Example Tweet 3 Answer: True/False/Irrelevant Instruction: Classify tweets as being in agreement (true), disagreement (false), or irrelevant with the belief that AI is [adjective].
Zero-shot CoT^b^	Classify tweets as being in agreement (true), disagreement (false), or irrelevant with the belief that AI is [adjective]. Let's go step by step.
Few-shot CoT	Tweet: Example Tweet 1 Thought: Example Explanation 1 Answer: True/False/Irrelevant Tweet: Example Tweet 2 Thought: Example Explanation 2 Answer: True/False/Irrelevant Tweet: Example Tweet 3 Thought: Example Explanation 3 Answer: True/False/Irrelevant Instruction: Classify tweets as being in agreement (true), disagreement (false), or irrelevant with the belief that AI is [adjective].

^a^AI: artificial intelligence.

^b^CoT: chain of thought.

This created a dataset of GPT-generated labels for all benchmark posts.

We defined the performance metric as weighted *F*_1_-score (w*F*_1_-score), calculated for the classification of each domain as in the equation below, where *a, b,* and *c* refer to the number of posts classified to each category by human annotations, and *True, False, Irrelevant,* refer to the *F*_1_-scores for each classification):



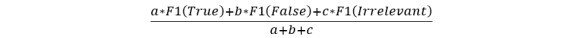



This compared the predicted (LLM) labels of “true,” “false,” and “irrelevant” for each domain to the true (researcher) labels for the same posts. Model performance was calculated using nonparametric bootstrapping of 1000 resamples with replacement from each domain’s n=150 annotated items, resulting in domain-specific mean w*F*_1_-score and 95% CIs [47]. Prompt ablation is reported in Figure S3 and Table S3 of Multimedia Appendix 1.

We prespecified a threshold of w*F*_1_-score>0.6 as the minimum acceptable agreement with human annotations. This threshold was selected based on the historic performance of sentiment models and emergent applications of LLMs, like GPT-3.5-Turbo for annotation [24,30,48], along with the inherent subjectivity of the described task.

GPT-3.5 Turbo was chosen for cost efficiency to label the full dataset (Table 1). Prompts with w*F*_1_-scores>0.6 were passed to the model for labelling of the full dataset (Multimedia Appendix 2). Verbatim prompt variations, along with model parameters (eg, version, temperature, and random seed), are specified in Multimedia Appendix 2.

### Data Curation

Data for this study were sampled from X, selected because of its status as a major site of health communications for health-related institutions, academia, companies, and interested individuals [49], with over 550 million monthly users in 2023 [50].

X posts from January 1, 2023 to December 5, 2023 – an 11-month period when ChatGPT site visits grew by 1 billion [51], were collected using AI-related keywords. Keywords included popular LLMs like “ChatGPT,” techniques such as “natural language processing,” and AI domains like “conversational AI,” along with the term “health.” For the full Boolean search string, refer to Figure S1 in Multimedia Appendix 1. Tweets in English were included. As LLM performance for sentiment classification tasks is variable for low- and mid-resource languages [52], consistency through English texts was opted for to avoid introducing variability in processing and interpretation of model annotations. In addition, a previous analysis of ChatGPT-related tweets from an overlapping time period found 72% were in English [11]. In total, 786,750 posts were selected, including original posts, retweets, and quoted tweets – posts responding to an original post.

Qualitative review of the sample identified posts shared from general users, news sources, academic institutions, health organizations, influencers – especially tech leaders, and private companies. Past estimates suggest between 9% to 15% of active X users have automated accounts [53]. This reflects the reality of social media as a site of information exchange between individuals, organizations, and even bots.

### Data Cleaning

For sentiment analysis, duplicates were removed from the raw dataset, leaving 394,533 posts.

For emotion analysis and topic modeling, we filtered out posts that only contained hashtagged text or contained 4 or more stock tickers (eg, $XXX). The following preprocessing steps were then applied: emoticons, usernames (starting with “@”), URLs, special characters, “retweet,” “QT” (quote tweet), and stock ticker patterns were removed. Multiple spaces, tabs, and newlines were collapsed into a single space. Duplicates were dropped again, leaving 268,347 posts (Figure S2 in Multimedia Appendix 1).

For topic modeling, the text was converted to lowercase. Tokenization was performed with spaCy (English model en_core_web_lg). We retained only alphabetic tokens, applied lemmatization and lowercasing, and removed tokens that were stopwords from the spaCy default list or had part of speech tags ADV, PRON, CCONJ, PUNCT, PART, DET, ADP, SPACE, NUM, or SYM. Common tokens were standardized, for example, “ml” to “machine learning.”

### Emotion Analysis

Compared with research on perspectives toward AI, the role of emotions in the expression and formation of these attitudes is understudied. Previous work has found that individuals with strong anger and less hope show more support for AI upon exposure to information sources [54]. To explore the emotional content of discussions, we used “Emollama-7b”—an open source LLM finetuned from LLaMA2-7B for affective analysis.

Emotion analysis used the model’s 11 recommended emotional classifications. This includes 6 emotions previously studied for their implication in decision-making, including judgments of science [55]. A previous benchmark found that Emollama-7b outperforms ChatGPT and GPT-4 for the emotion classification task: “Given a tweet, classify it as ‘neutral or no emotion’ or as one, or more, of eleven given emotions (anger, anticipation, disgust, fear, joy, love, optimism, pessimism, sadness, surprise, trust) that best represent the mental state of the tweeter” [32,56]. This identical prompt was used to instruct Emollama-7b to label n=268,347 X posts. Default parameters were used based on the model downloaded from Hugging Face [57]. We clarify that “trust” in the emotion analysis refers to an affective state label from the emotion model and is conceptually distinct from attribute‑level trustworthiness of health AI measured in the sentiment analysis.

### Topic Modeling

To characterize the thematic dimensions of health AI discussed online, we applied latent Dirichlet allocation (LDA), an unsupervised probabilistic topic-modeling approach in which documents (X posts) are represented as mixtures of latent topics, and topics as distributions over words [58]. LDA has been widely used to summarize large-scale health-related social media corpora [59-62].

We implemented LDA using the Gensim LDAMulticore function in Python. Tokens occurring more than 10 times across the corpus and in fewer than 50% of posts were retained in the dictionary to reduce sparsity and remove highly ubiquitous terms. Candidate models ranging from k=3 to k=12 topics were evaluated using the Cv coherence metric. For each model, the training procedure used 10 passes over the corpus and 50 iterations. Hyperparameters (alpha and beta) were retained at default values.

Because LDA inference is stochastic, we assessed topic stability by refitting the k=10 model across 4 random initialization seeds and comparing the consistency of high-probability keywords and overall thematic structure. Although some variation in keyword ordering occurred across runs, the main thematic groupings were consistent. The final model (k=10) was selected based on coherence trends across candidate models and qualitative interpretability, balancing thematic granularity with semantic distinctiveness. Furthermore, 3 researchers (LMW, ZW, and LL) then iteratively refined topic labels by synthesizing the top 10 highest-probability words per topic and validating each label against random samples of posts [63]. Topics were subsequently summarized by WHO region and emotional distribution.

### In-Depth Analysis of Global Public Confidence in Health AI

GPT-3.5 Turbo labeled sentiments and health AI confidence in X posts. After cleaning, 394,533 posts were submitted to GPT-3.5 Turbo for sentiment analysis. Due to OpenAI’s policy prohibiting discussions of violence, illegal activity, and adult content [64], several thousand explicit posts were excluded, resulting in a labeled dataset of 388,009. Posts without a GPT label were excluded from the analysis of each confidence domain.

GPT-labeled posts were grouped by WHO region based on user location, with excluded areas (eg, Hong Kong and Vatican City) added as needed (Table S1 in Multimedia Appendix 1). The top 5 countries with the most posts in each region are listed in Table S2 in Multimedia Appendix 1. Posts annotated as “positive,” “neutral,” and “negative” in sentiment were mapped to 1, 0, and –1, respectively. Averages were calculated as arithmetic means by WHO region, dividing the number of posts in each classification by the total number of posts in each region. 95% CIs are reported using a nonparametric bootstrap with n=1000 resamples. Reach was analyzed as the number of unique users who saw a post. This metric is provided from Meltwater’s (Meltwater US Holdings Inc) application program interface [65], which was used to extract posts from X based on our search criteria. Analyses and visualizations were conducted in Python (v3.11.8).

### Ethical Considerations

This study analyzed publicly available posts from X (formerly Twitter) and does not constitute human subjects research, as no private or identifiable information was collected and no interaction with individuals occurred. Accordingly, formal ethical review and informed consent were not required. All data were accessed through an authorized third-party data provider and handled in accordance with X’s terms of service. No personally identifiable information was reported; all analyses were conducted at the aggregate level, and no individual users are identified or identifiable in the manuscript or supplementary materials. As this study did not involve human participants, compensation was not applicable.

## Results

### Sentiment Analysis

Frequency and overall sentiment of posts by WHO region are summarized in Table 3. In total, 42 countries shared over 500 posts in the study period. Geographically, most posts came from the Region of the Americas (AMR), accounting for 121,201 (31.38%) posts, followed by the European Union with 57,234 (14.82%) posts. However, a large portion of posts (n=153,289, 39.68%) came from users who did not disclose their location on X.

**Table 3:** Sentiments about health artificial intelligence on X from January 1 to December 3, 2023. Percentages are calculated using the n of each region as the denominator.

		Overall (n=386,287)		AFR^a^ (n=10,624)		AMR^b^ (n=121,201)		EMR^c^ (n=6375)		European Union (n=57,234)		SEAR^d^ (n=24,888)	WPR^e^ (n=12,676)	Undisclosed (n=153,289)
**Positive**														
	n		252,082		7179		79,284		4736		37,848	16,974	7608	98,453
	Sample proportion^f^,% (95% CI)		65.26 (65.11-65.4)		67.57 (66.63-68.48)		65.42 (65.16-65.67)		74.29 (73.13-75.34)		66.13 (65.76-66.5)	68.2 (67.61-68.74)	60.02 (59.2-60.85)	64.23 (65.11-65.4)
**Negative**														
	n		36,505		721		11,565		227		4639	930	1217	17,206
	Sample proportion, % (95% CI)		9.45 (9.36-9.54)		6.79 (6.31-7.26)		9.54 (9.37-9.71)		3.56 (3.11-4)		8.11 (7.87-8.32)	3.74 (3.51-3.98)	9.60 (9.1-10.07)	11.22 (11.07-11.38)
**Neutral**														
	n		97,700		2724		30,352		1412		14,747	6984	3851	37,630
	Sample proportion, % (95% CI)		25.29 (25.15-25.43)		25.64 (24.83-26.53)		25.04 (24.79-25.29)		22.15 (21.08-23.12)		25.77 (25.4-26.11)	28.06 (27.47-28.59)	30.38 (29.55-31.18)	24.55 (24.34-24.77)
Mean sentiment score (SD; 95% CI)			0.5561 (0.6600; 0.5581-0.5602)		0.6079 (0.6117; 0.5960-0.6194)		0.5587 (0.6614; 0.5551-0.5625)		0.7073 (0.5275; 0.6947-0.7194)		0.5802 (0.6369; 0.5749-0.5856)	0.6446 (0.5512; 0.6377-0.6515)	0.5042 (0.6648; 0.4924-0.5151)	0.5300 (0.5267-0.5335)

^a^AFR: African Region.

^b^AMR: Region of the Americas.

^c^EMR: Eastern-Mediterranean Region.

^d^SEAR: South-East Asia Region.

^e^WPR: Western Pacific Region.

^f^Sample proportion: Number of posts/total.

The majority of posts were positive (n=252,082, 65.26%), followed by neutral (n=97,700, 25.29%), and negative (n=36,505, 9.45%) about health AI. The sentiment distribution of WHO regions revealed significant differences (*χ*^2^=2274.1, *P*<.001). The Eastern Mediterranean Region (EMR) was most positive about health AI, with nearly three-quarters of posts expressing positive sentiments. While the Western Pacific Region (WPR) had the lowest sentiment score of 0.50, driven by a higher percentage of neutral sentiment posts (3851/12,676, 30.38%), posts from an undisclosed region shared the highest proportion of negative comments about health AI, at 11.22% (37,630/153,289).

### Confidence in Health AI

**Figure 1:**
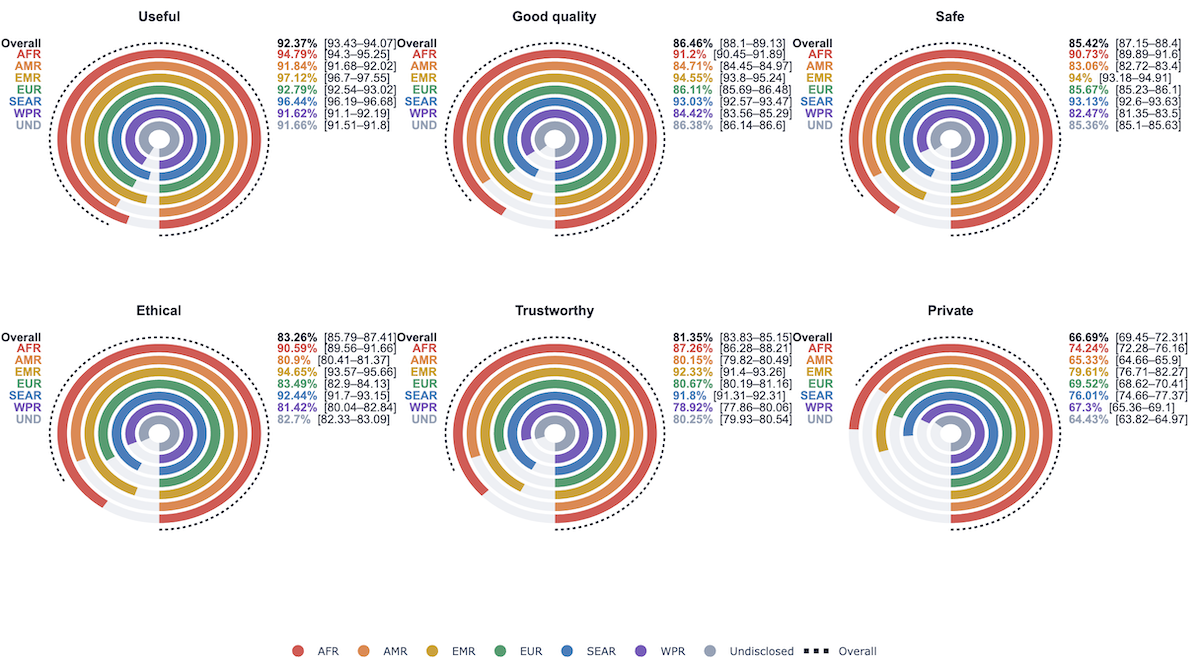
Sample proportion (95% CI) of X posts that expressed attitudes in favor of health AI attributes, grouped by WHO region (January 1 to December 3, 2023). AFR: African Region; AMR: Region of the Americas; EMR: Eastern-Mediterranean Region; EUR: European Union; SEAR: South-East Asia Region; WPR: Western Pacific Region; UND: Undisclosed.

The majority of posts expressed attitudes in favor of health AI. “Privacy” showed the biggest divide in opinions for or against the technology, with 45,977/68,943 (66.69%; 95% CI 66.34%-67.02%) of privacy-related posts affirming health AI’s respect for privacy, while 22,966/69,943 (33.31%; 95% CI 32.98%-33.66%) of related posts were classified as indicating health AI threatened privacy. This concern was highest in AMR and undisclosed regions, with averages in support of privacy falling significantly below the overall sample proportion. In AMR, 14,803/22,660 (65.33%; 95% CI 64.7%-65.96%) of related posts supported health AI’s respect for privacy, while 17,285/26,828 (64.43%; 95% CI 63.88%-64.99%) of posts expressed the same belief in undisclosed regions (Figure 1). Trustworthiness was the next most divided topic, but 136,700/168,041 (81.35%; 95% CI 81.16%-81.53%) of trust-related posts still voiced trust toward health AI. By comparison, the usefulness of health AI was nearly unanimous. There were 291,457 in favor of usefulness out of 315,528 related posts, totaling 92.37% (95% CI 92.28%-92.47%) of posts in the overall sample in support of health AI’s usefulness. Support within EMR was significantly higher at 5321/5479 (97.12%; 95% CI 96.7%-97.57%) of relevant posts.

EMR reported the highest percentage of posts in favor of health AI across all 6 domains. For “usefulness,” “quality,” “safety,” and “trust,” WPR had the lowest percentage of posts in favor, but rose above the overall sample proportion for “privacy.” AMR consistently voiced lower support for health AI concepts than the overall sample. Full results of the sentiment analysis are reported in Table S5 in Multimedia Appendix 1.

### Emotion Analysis and Topic Modeling

Considering the 11 possible emotions explored in discussions of health AI, out of the total sample, 224,386 (83.62%; 95% CI 83.48%-83.76%) posts contained “optimism.” This was followed by “anticipation,” found in 165,300 (61.6%; 95% CI 61.42%-61.77%) posts. “Optimism” was nearly 10 percentage points higher than the overall sample in the South-East Asia Region (SEAR), found in 15,860 posts (91.79%; 95% CI 91.41%-92.2%) and EMR in 4279 (91.63%; 95% CI 90.79%-92.4%) posts. Moreover, 7563 (80.84%; 95% CI 80.07%-81.65%) posts from WPR contained optimism, which was the lowest regional proportion. WPR expressed the highest proportion of all negative emotions: pessimism, disgust, sadness, anger, and fear, at a rate consistently above the overall sample. This included 3021 (32.29%; 95% CI 31.34%-33.23%) posts from the region that contained pessimism. Full emotional results are available in Table S7 in Multimedia Appendix 1. Posts with “anticipation” reached the most accounts on average (8604.54, 95% CI 8099.95-9136.3), followed by trust (8596.68, 95% CI 7396.50-10,074.38), and fear (7721.15, 95% CI 6455.61-9146.52).

**Figure 2:**
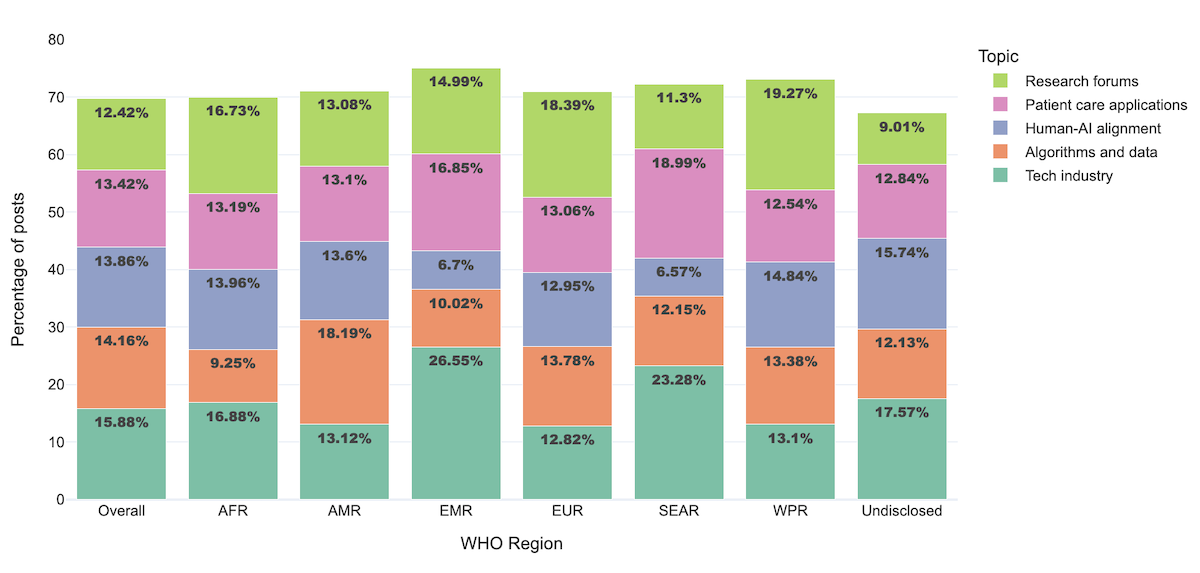
Distribution of top 5 health artificial intelligence topics on X by World Health Organization region, January 1 to December 3, 2023. AFR: African Region; AI: artificial intelligence; AMR: Region of the Americas; EMR: Eastern-Mediterranean Region; EUR: European Union; SEAR: South-East Asia Region; WHO: World Health Organization; WPR: Western Pacific Region.

The 10 topics generated by the LDA topic model reflected the diversity of considerations when it comes to health AI, spanning commercial excitement from product developments to ethical considerations of responsible AI development. From the top 5 most popular topics (Table 4), the first was “tech industry,” characterized by terms like “business,” “digital,” and “innovation.” These tweets focused on the commercial potential of the digital health revolution, including direct marketing from industry stakeholders. This topic was most common within EMR, found in 3594 (26.55%; 95% CI 25.33%-27.74%) local posts, followed by 11,429 (23.29%; 95% CI 22.65%-23.91%) within SEAR, and 51,956 (17.43%; 95% CI 17.35%-17.8%) within undisclosed regions. The second most popular topic was “algorithms and data,” which frequently used the terms “datum,” “model,” and “patient.” Review of sample posts found discussions of the use of personal health data for health prediction and disease models, and it was most prominent in AMR, which shared 47,910 (18.19%; 95% CI 17.92%-18.44%) related posts of discussions within the region. This was followed by “human-AI alignment,” addressing AI’s alignment with human ethics and values. Notably, 40.25% (14,966/37,184) of these discussions mentioned “mental health,” along with terms like “help” and “people.” “Patient care applications” examined specific AI-driven health solutions and products, such as the creation of drug treatments and diagnostic abilities, referencing words like “treatment,” “improve,” “patient,” and “care.” Finally, “research forums” highlighted emerging research opportunities like conferences and academic positions using words such as “join,” “digital,” “global,” and “work.” Interest in research was predominant within WPR, which shared n=6950 (19.27%; 95% CI 18.5%-20.07%) related posts from the region, European Union at n=19,192 (18.39%; 95% CI 18.01%-18.8%) posts, and the African Region with n=3657 (16.75%; 95% CI 15.92%-17.6%) posts (Figure 2).

**Table 4:** The 5 most frequent topics in health AI-related discussions on X, January 1 to December 3, 2023. Top 10 most probable and salient terms are reported per topic.

Topic name	n (%; 95% CI)	Probable terms	Salient terms	Examples
Tech industry	42,399 (15.88; 15.67-15.94)	business, ChatGPT, public, digital, ethical, social, technology, education, system, and need	industry, future, technology, revolutionize, innovation, machine learning, finance, transform, potential, and blockchain	“Explore the world of AI chatbots across industries! From e-commerce to healthcare, these intelligent tools are reshaping businesses. Discover how they align with industry KPIs and stay updated on the latest innovations. Dive in!” “As an entrepreneur, I learnt how to use technology to create real impact & pivot my company to be ahead of the curve. Being among the founders of top AI, education & healthcare companies, & understanding the new age findings in AI, AR/VR, robotics & medicine was super insightful.”
Algorithms and data	37,700 (14.05; 13.92-14.19)	datum, model, patient, medical, care, generative, system, new, clinical, and machine learning	patient, datum, medical, improve, treatment, model, care, disease, outcome, and diagnosis	“Our generative AI (really a deconstructed neural network-based expert system model built on established public scientific evidence) enables both patients and clinicians to increase their connection and collaboration leading to better and more deeply personalized healthcare.” “Excellent article highlighting issues around AI in health care. Notes a number of challenges: ‘a major issue is the relative scarcity of publicly available data sets in medicine.’ Reproducibility issues that haunt health-care AI”
Human-AI^a^ alignment	37,184 (13.86; 13.72-13.99)	mental, people, need, human, help, ChatGPT, support, work, good, and time	mental, people, need, talk, help, ChatGPT, good, therapy, diaspora, and feel	Are you the type of person who wants to stick with the old techniques? If so, It's better if you start learning how to flip the burgers, or wait, even AI will replace these jobs‚ AI is on absolute fire! “me: for the good of mankind we need to beat up anyone in AI development with hammers Everyone around me: oh so you hate technology huh! People developing AIs”
Patient care applications	33,850 (12.6; 12.48-12.73)	patient, care, medical, treatment, improve, disease, diagnosis, help, technology, and doctor	digitalhealth, doctor, healthtech, chatgpt, patient, care, healthit, news, telemedicine, and medicine	“Artificial intelligence is having a MAJOR breakthrough within the healthcare industry right now! It’s playing a huge role in the discovery of new drugs, triaging, and fighting specific resistances against drugs.” “The predictive capabilities of AI tools save invaluable time, allowing #healthcare professionals to focus on what truly matters - their patients. By improving diagnostic processes, #patientsatisfaction, and overall #health outcomes are bound to reach new heights.”
Research forums	33,198 (12.37; 12.25-12.49)	research, join, global, digital, work, science, innovation, discuss, project, and future	join, research, discuss, global, register, event, conference, science, university, and discussion	“As we mark #BreastCancerAwarenessMonth, check out this study on AI’s transformative role in same-day diagnostics from #AHRQ’s Digital Healthcare Research Program 2022 Year in Review.” “Learn all about federated learning in healthcare projects at AI Meetup next week! Amii’s Payam Mousavi leads a panel discussion feat. Ross Mitchell, Ruchika & Neeraj Kumar, three co-authors of a recently published study in @NatureComms”

^a^AI: artificial intelligence.

**Figure 3:**
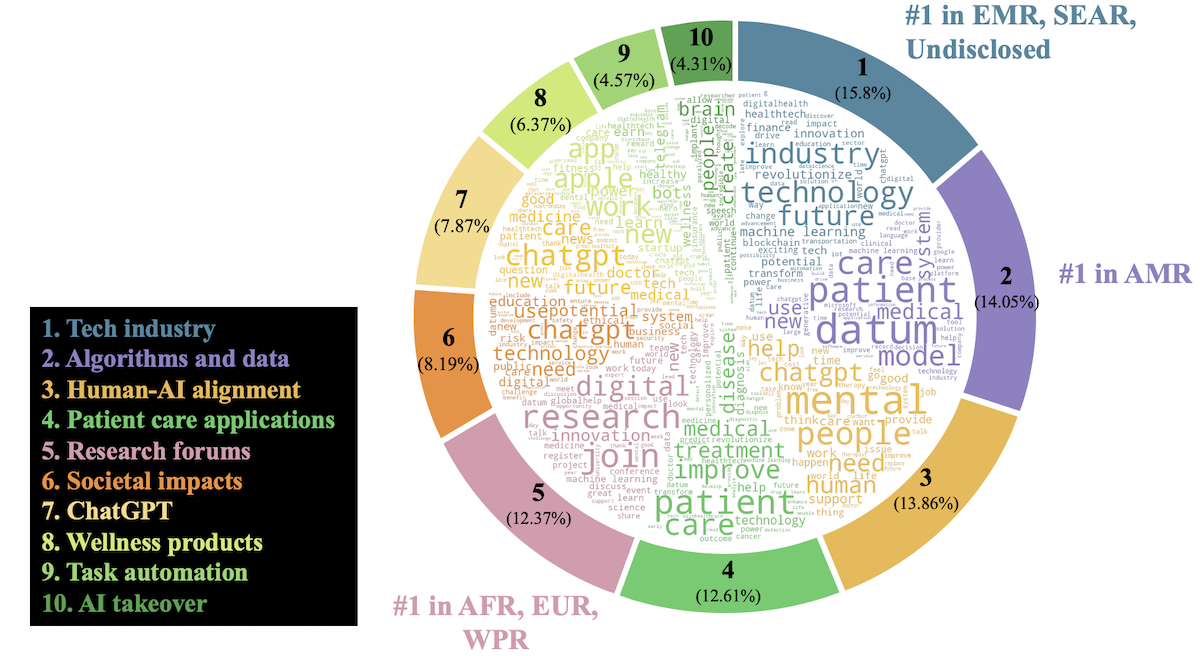
Ten topics and respective keywords in health artificial intelligence–related discussions on X, January 1 to December 3, 2023. “#1” denotes the most frequent topic in a World Health Organization region. AFR: African Region; AI: artificial intelligence; AMR: Region of the Americas; EMR: Eastern-Mediterranean Region; EUR: European Union; SEAR: South-East Asia Region; WPR: Western Pacific Region.

For all 10 topics, we report the top terms with topic word probabilities and example posts in Table S7 in Multimedia Appendix 1 to support interpretability and reproducibility of labels. The relative frequency of keywords characterizing each of the 10 topics is plotted in Figure 3, representing key terms contributing to the topic model.

**Figure 4:**
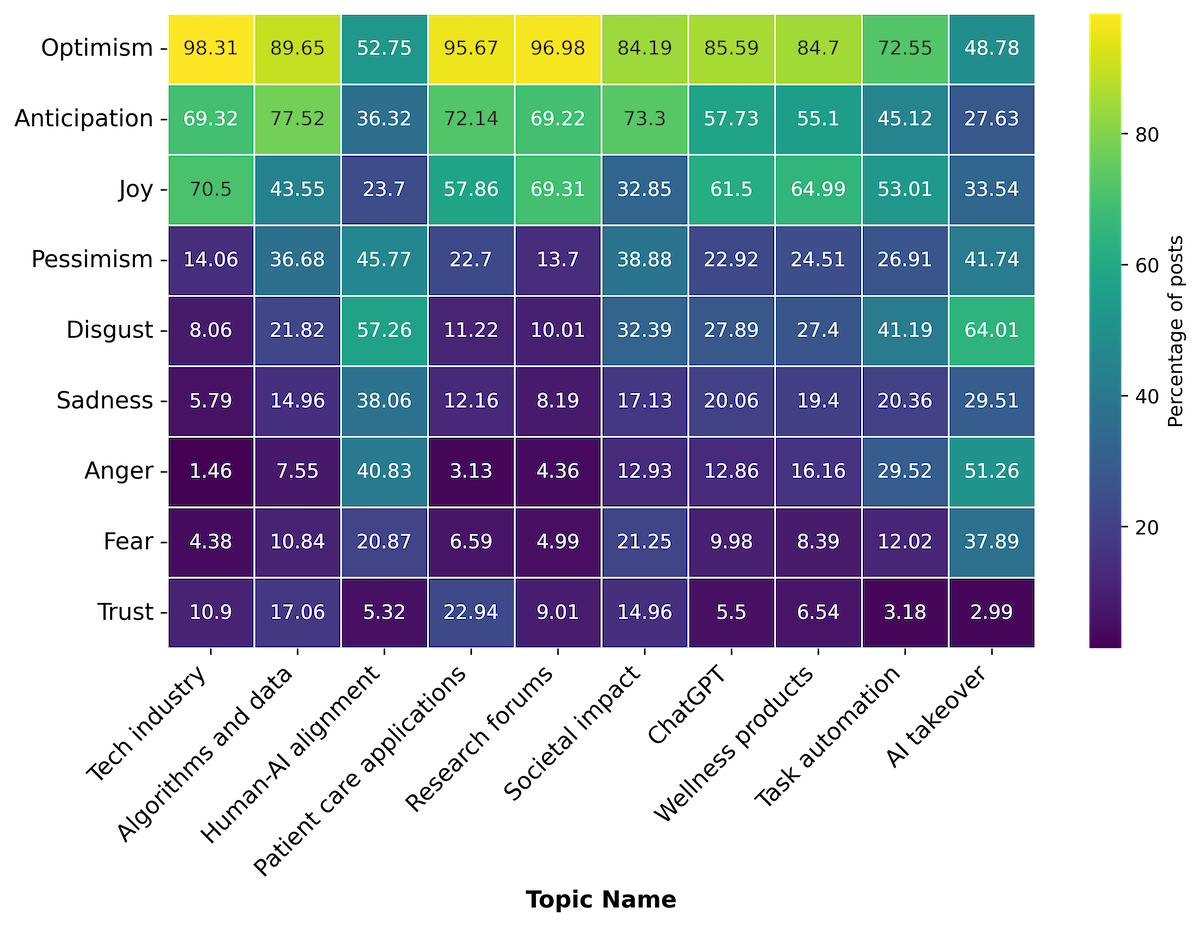
Emotional content of 10 topics found in discussions about health artificial intelligence on X, January 1 to December 3, 2023. AI: artificial intelligence.

Topics were differently emotionally charged. Each post could be represented by multiple emotions. In “tech industry,” 41,816 (98.31%; 95% CI 98.19%-98.43%) post contained optimism. Similarly, in “research forums,” 32,240 (96.98; 95% CI 96.8%-97.17%) posts contained optimistic language, respectively. Despite also showing high rates of optimism, pessimism was found in 7793 (38.87%; 95% CI 38.23%-39.49%) posts in “societal impacts,” along with 13,874 (36.68%; 95% CI 36.17%-37.2%) in “algorithms and data,” reflecting mixed outlooks on these issues. By comparison, 21,648 posts in “human-AI alignment” and 6948 posts in “AI takeover” contained disgust, making it more prevalent than optimism in both topics (Figure 4). Anger was also common in these topics; in “human-AI alignment,” 15,019 (40.83%; 95% CI 40.31%-41.3%) posts contained anger, while in “AI takeover,” 5564 (51.27%; 95% CI 50.26%-52.17%) did.

“Love,” “surprise,” and “neutral” (no emotion identified) were in less than 1% of posts and omitted from Figure 4.

## Discussion

### Principal Findings

The rapid rise in public attention catalyzed by generative AI—particularly following the release of ChatGPT—coincided with a marked expansion in the scale and visibility of public discourse on health AI in 2023. In this analysis of posts on X using LLM-enabled methods, discussions were predominantly favorable across regions, with perceived usefulness emerging as the strongest driver of confidence. At the same time, privacy was consistently the least trusted dimension, indicating that enthusiasm for the potential of health AI is accompanied by persistent concern about data governance. Emotional patterns reinforced this tension; optimism dominated conversations about innovation, patient care, and research, whereas fear and anger were concentrated in discussions of human-AI alignment and AI takeover. We also identified significant regional variation in these narratives, with EMR and SEAR showing more innovation-oriented and optimistic discussions, while WPR, the European Union, and AMR placed greater emphasis on privacy, ethics, and alignment. Taken together, these findings contribute to the characterization of how health AI is framed and viewed online, showing that public confidence in health AI is not uniform, but multidimensional, regionally shaped, and defined by a balance between promise and concern.

### Regional and Thematic Patterns in Health AI Discourse

We found 65% (252,082/386,287) of posts to be positive in sentiment about health AI. This figure is moderately higher but directionally consistent with attitudes toward health AI found on other social media and surveys. A previous study of X and Reddit posts from 2021 to 2024 showed 55% (566/1022) expressed positive sentiments toward AI in medical imaging [22], while 58% (7775/13,502) of patients in a 2023 global survey were positive about AI’s integration into health care [66]. The somewhat higher positivity in our sample may reflect the study time period’s overlap with significant model developments that generated excitement online. Following the release of ChatGPT in late 2022, conversation volume surged from zero related tweets to 550,000 per day by late January 2023, with average sentiment consistently more than 50% positive in the first few months [11]. Studies including data before and after 2023 may reflect stabilization to a more even distribution between sentiments than we observed. The discussion focus on industry and commercial use cases parallel results from a study of social media from China (Weibo [Sina Corporation]) [10], a region largely excluded from this study’s sample on X. This suggests global platforms are commonly used to discuss and promote health AI business, followed by discussions of AI’s impact and alignment with society [10]. In the media’s portrayal of general AI, business tends to be the most common positive angle [67], which we also observed in the near-unanimous level of optimism found in posts discussing the health AI tech industry.

We found enthusiastic attitudes were most prevalent in EMR and SEAR, where positive sentiments and optimistic emotions were significantly higher than in other regions. By comparison, EMR and SEAR rarely mentioned human-AI alignment, which was more popular in WPR and undisclosed regions. The high prevalence of tech industry–focused discussions, at 27% in EMR and 23% in SEAR, aligns with a previous study of news media from the same countries that are primarily represented in the EMR and SEAR samples, the United Arab Emirates, and India, respectively. Nearly 25% of coverage from the United Arab Emirates focused on industry expansion and economic initiatives, while 21% of articles from India discussed startups [68]. Both countries’ newspapers did not mention regulations, privacy, or ethics [68]. A potential feedback loop may exist in which supportive media narratives and social media discourse mutually reinforce optimistic narratives about health AI adoption, while simultaneously minimizing discussion of specific concerns more prevalent in other regional media.

On the other hand, social media posts from mostly Western countries in WPR, the European Union, and AMR expressed less confidence in the technology, with greater concern for privacy, ethics, and AI alignment. Sentiment toward health AI was less positive than EMR and SEAR. Emotions of disgust, pessimism, and fear were elevated in posts from the region, mirroring an increasing trend of fear-invoking headlines trend from local newspapers [68]. In the European Union and WPR, discussions about research, including academic funding, jobs, and conferences, outnumbered mentions of industry. This “responsibility” focus is plausibly explained by regional contexts. In the study period, the European Union was where the first legal framework on AI, the AI Act, was being deliberated [69], while AMR’s topical focus on algorithms and data reflects longstanding public concerns over data use and privacy in the United States [8]. Yet across all regions, privacy was the most consistent concern, substantially present even in EMR and SEAR with otherwise positive sentiment and optimistic emotional tone. This convergence suggests that privacy is a near-universal dimension of public unease about health AI that persists regardless of the broader narrative orientation of a region’s discourse. In this case, social media represents an important middle ground of social expression between traditional media sources, which in some regions contain no mention of AI-related privacy issues [68], and direct surveys of patients, which suggest concern about health AI privacy is above 50% [70]. Where in-depth surveying is limited or not feasible, social media can be analyzed to surface latent concerns that traditional media underreports. The privacy concerns found in this study suggest data governance is a cross-cutting barrier to health AI adoption, even in regions where the broader online narrative appears favorable.

Taken together, these regional patterns demonstrate social media as a real-time, decentralized information environment where competing narratives about AI unfold, distinct from both institutional media and individually held viewpoints. A single global communication strategy for health AI is therefore unlikely to be effective, and instead must address how the proliferation of social media discourse surrounding AI might shape real-world instances of public trust in health technologies. The concentration of high-arousal negative emotions around existential AI narratives is particularly consequential; negative, emotionally charged content is associated with greater spread of posts, including misinformation [16,71], which could explain why posts using fear to discuss health AI reached close to 8000 users on average. Drawing on previous experiences with vaccine hesitancy, online misinformation is associated with and can affect health technology beliefs and adoption intention [72,73], including a negative relationship between misinformation consumption and COVID-19 vaccine uptake [74,75]. Policymakers and health authorities should consider integrating AI-enabled social listening into health technology assessment, risk communication, and regulatory monitoring to proactively anticipate emerging concerns and enable more responsive, publicly aligned health AI governance.

### Practical Implications of LLMs

This study evaluated LLM-based annotation for health-related social media analysis using the AI Usage Consideration Checklist (Multimedia Appendix 3). In terms of accuracy of information, a few-shot CoT approach frequently outperformed previous evaluations of off-the-shelf sentiment tools and zero-shot LLMs, and achieved comparable performance to fine-tuned models on the same metric (w*F*_1_-score) [24,30]. This suggests that few-shot CoT prompting can meaningfully improve annotations for social media analysis, an area where traditional sentiment tools often perform poorly [26,30]. GPT-4 outperformed GPT-3.5-Turbo across annotation tasks, achieving w*F*_1_-scores above 0.7, and continued model development suggests further performance gains are likely.

Regarding usability, the LLM pipeline was accessible to noncomputer science researchers via natural language instructions and simple Python scripts, lowering the barrier to adoption for public health institutions with otherwise limited machine learning capacity. LLMs also enabled annotation of over 300,000 posts, a scale not attained through traditional manual content analysis on the same topic [10,22].

### Limitations and Future Research

This study has several limitations. First, although all annotation tasks achieved w*F*_1_-scores above 0.6, performance varied across classification constructs, and complex dimensions, such as trust in AI, remain challenging given the lack of definitional consensus even among human experts [76]. Our analysis distinguished between perceived trustworthiness and affective trust [77] as a pragmatic solution in the absence of an established framework for confidence in health AI; however, this approach is operationalized for scalable social listening and should be interpreted as a descriptive monitoring framework using constructs informed by the literature, rather than a validated psychometric instrument. Misclassification is possible due to LLM tendencies to exaggerate interpretations of positive or negative sentiments [26], along with classification challenges when short texts contain sarcasm or irony, which both humans and language models struggle with [47]. To mitigate these risks, we implemented a structured annotation framework with predefined categories, applied benchmarking thresholds, and reported attribute-level patterns rather than aggregate polarity scores alone. Future work should incorporate formal interannotator validation, larger benchmarks, and model comparisons. Constructs should also be refined and validated against survey-based or experimental measures to strengthen conceptual alignment and elucidate relationships between the online health AI landscape and offline attitudes to such technologies. Third, our analysis was restricted to global English-language posts on X, which may underrepresent non–English-speaking populations and regions where the platform is restricted. However, this focus reflects the empirical context of data collection in 2023, immediately following the release and widespread uptake of predominantly English-language generative AI systems, during which early public discourse was largely English-language dominated [11]. As AI systems become increasingly multilingual and globally embedded, future studies should expand to non-English datasets and additional platforms to enhance cross-cultural validity and representativeness. Finally, model performance and construct mapping are time- and context-specific, and both public discourse and model behavior may shift over time. Nevertheless, this adaptability is also a strength; computational approaches can be rerun and recalibrated as language and technology evolve. We encourage periodic re-evaluation of model performance and updated benchmarking datasets.

This study advances previous social media analyses of health AI [10,22] by moving beyond basic sentiment classification to enable multidimensional characterization of public confidence across 42 countries during a period of rapid growth in AI-related online activity. The integration of LLM-based stance and emotion annotation with LDA topic modeling allowed for context-sensitive classification of complex constructs at scale. These findings establish a reproducible framework for scalable surveillance of online attitudes in digital health research that can be extended to other health technologies as they enter public discourse, and recalibrated as the language used to discuss AI continues to evolve. For policymakers, the regional and thematic granularity of discourse data provides detailed analysis about what information online populations are being exposed to and how digital ecosystems respond to changing developments in both technology and regulation. Among health system leaders and communicators, real-time social listening of this kind can anticipate concerns before implementation, including in regions where direct surveying is limited, to understand what aspects of the technology are viewed positively in popular discourse and which still face resistance.

## Appendix

Multimedia Appendix 1 Description of methodology and full results of all analyses.

Multimedia Appendix 2 GPT prompts and parameter settings.

Multimedia Appendix 3 AI usage consideration checklist.

## Abbreviations

AI: artificial intelligence
AMR: Region of the Americas
CoT: Chain of Thought
EMR: Eastern-Mediterranean Region
LDA: latent Dirichlet allocation
LLM: large language model
NLP: natural language processing
SEAR: South-East Asia Region
wF1-score: weighted F1-score
WHO: World Health Organization
WPR: Western Pacific Region

## Data Availability

The social media data analyzed in this study cannot be made publicly available due to licensing and contractual restrictions governing access to the platform data. Researchers seeking to reproduce the findings should obtain independent access to X data through authorized third-party providers or formal research agreements with the platform. The full search strategy and filtering criteria used to construct the dataset are described in Multimedia Appendix 1 to support methodological transparency and reproducibility.
