# Supplementary material for: Global Sentiment Toward Health AI at the Dawn of the ChatGPT Era: Empirical Analysis of Twitter (X) Discourse
Source: J Med Internet Res. 2026 May 5;28:e80346. doi: 10.2196/80346 (PMC13187703; doi:10.2196/80346)
Supplement: Multimedia Appendix 1 [file jmir_v28i1e80346_app1.doc]

**Methods**

Data Extraction

| (AI OR "artificial intelligence" OR "GPT" OR ChatGPT OR NLP OR "Natural Language Processing" OR "Machine Learning" OR "Deep Learning" OR "transformer" OR "Bert" OR "Midjournery" OR "Stable Diffusion" OR "Generative AI" OR "AGI" OR "Conversational AI" OR "Text Generation") AND Health |
| --- |

**Supplemental Figure 1. Keyword search string used in Meltwater tweet extraction.**

**LLM Validation**

**
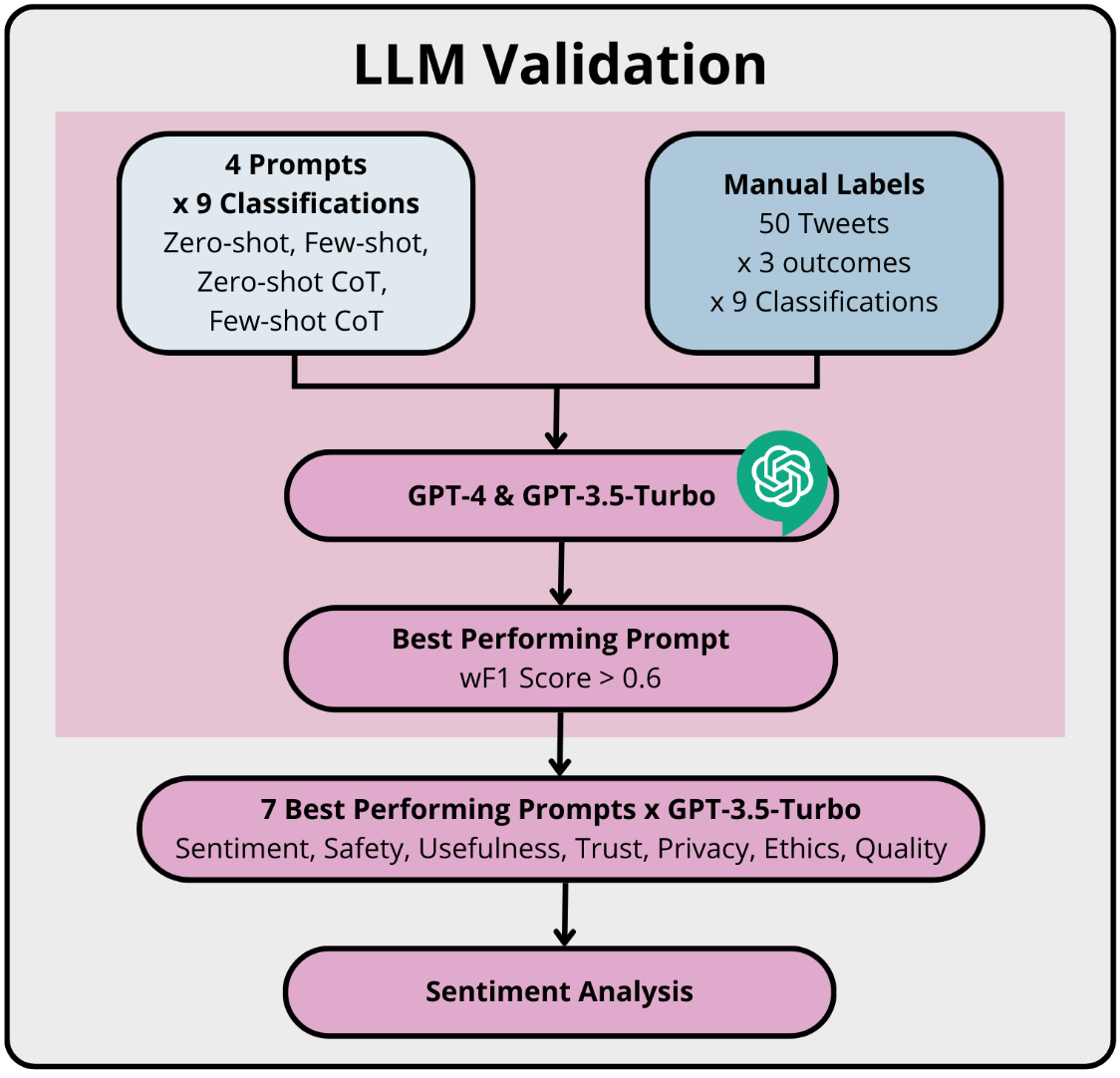
**

**Supplemental Figure 2. LLM Validation and Prompt Ablation.** Four prompting techniques (zero-shot, few-shot, zero-shot CoT, few-shot CoT) with two model engines were compared against human labels for nine classifications (overall sentiment and eight health AI domains). Best-performing prompts (wF1 > 0.06) was used with GPT-3.5 Turbo for full dataset labeling.

| African Region | "Algeria", "Angola", "Benin", "Botswana", "Burkina Faso", "Burundi", "Cameroon", "Cabo Verde", "Central African Republic", "Chad", "Comoros", "Ivory Coast", "Democratic Republic of the Congo", "Equatorial Guinea", "Eritrea", "Ethiopia", "Gabon", "Gambia", "Ghana", "Guinea", "Guinea-Bissau", "Kenya", "Lesotho", "Liberia", "Madagascar", "Malawi", "Mali", "Mauritania", "Mauritius", "Mozambique", "Namibia", "Niger", "Nigeria", "Republic of the Congo", "Reunion" ,"Rwanda", "Sao Tome and Principe", "Senegal", "Seychelles", "Sierra Leone", "Saint Helena", "South Africa", "South Sudan", "Eswatini", "Togo", "Uganda", "Tanzania", "Western Sahara", "Zambia", "Zimbabwe" |
| --- | --- |
| Region of the Americas | "Antigua and Barbuda", "Anguilla", "Argentina", "Aruba", "Bahamas", "Barbados", "Belize", "Bolivia", "Bonaire, Saint Eustatius and Saba " ,"Bermuda", "Brazil", "British Virgin Islands", "Canada", "Cayman Islands", "Chile", "Colombia", "Costa Rica", "Cuba", "Curacao" ,"Dominica", "Dominican Republic", "Ecuador", "El Salvador", "French Guiana" ,"Grenada", "Guadeloupe" ,"Guatemala", "Guyana", "Haiti", "Honduras", "Jamaica", "Montserrat", "Mexico", "Nicaragua", "Panama", "Paraguay", "Peru", "Puerto Rico" ,"Saint Barthelemy" ,"Saint Kitts and Nevis", "Saint Lucia", "Saint Vincent and the Grenadines", "Sint Maarten", "Suriname", "Trinidad and Tobago", "Turks and Caicos Islands" ,"United States", "Uruguay", "U.S. Virgin Islands", "Venezuela" |
| European Region | "Albania", "Andorra", "Armenia", "Austria", "Azerbaijan", "Belarus", "Belgium", "Bosnia and Herzegovina", "Bulgaria", "Croatia", "Cyprus", "Czechia", "Denmark", "Estonia", "Finland", "France", “Greenland", "Georgia", "Germany", "Gibraltar", "Greece", "Guernsey" , "Hungary", "Iceland", "Ireland", "Isle of Man", "Israel", "Italy", "Jersey", "Kazakhstan", "Kosovo", "Kyrgyzstan", "Latvia", "Liechtenstein", "Lithuania", "Luxembourg", "Malta", "Moldova", "Monaco", "Montenegro", "Netherlands", "North Macedonia", "Norway", "Poland", "Portugal", "Romania", "Russia", "San Marino", "Serbia", "Slovakia", "Slovenia", "Spain", "Sweden", "Svalbard and Jan Mayen" , "Switzerland", "Tajikistan", "Turkey", "Turkmenistan", "Ukraine", "United Kingdom", "Uzbekistan", "Vatican" |
| Eastern Mediterranean Region | “Afghanistan", "Bahrain", "Djibouti", "Egypt", "Iran", "Iraq", "Jordan", "Kuwait", "Lebanon", "Libya", "Morocco", "Oman", "Pakistan", "Palestinian Territory", "Qatar", "Saudi Arabia", "Somalia", "Sudan", "Syria", "Tunisia", "United Arab Emirates", "Yemen" |
| South East Asia Region | "Bangladesh", "Bhutan", "North Korea", "India", "Indonesia", "Maldives", "Myanmar", "Nepal", "Sri Lanka", "Thailand", "Timor Leste" |
| Western Pacific Region | "American Samoa", "Australia", "British Indian Ocean Territory" , "Brunei", "Cambodia", "China", "Cook Islands", "Fiji", "French Polynesia", "Hong Kong" , "Japan", "Kiribati", "Laos", "Macao", "Malaysia", "Marshall Islands", "Micronesia", "Mongolia", "Nauru", "New Caledonia", "New Zealand", "Niue", "Norfolk Island" ,"Northern Mariana Islands" , "Palau", "Papua New Guinea", "Philippines", "Samoa", "Singapore", "Solomon Islands", "South Korea", "Taiwan", "Tonga", "Tuvalu", "Vanuatu", "Vietnam" |

**Supplemental Table 1.** Countries grouped by WHO region. Categorizations are according to official WHO guidelines, with missing regions added based on geographic location.

| **WHO Regions** | **Country** | **Counts** | **Proportion** |
| --- | --- | --- | --- |
| African Region | Nigeria | 3,596 | 33.71 |
| African Region | Kenya | 1,628 | 15.26 |
| African Region | South Africa | 1,476 | 13.83 |
| African Region | Tanzania | 729 | 6.83 |
| African Region | Cameroon | 601 | 5.63 |
| Eastern Mediterranean Region | Pakistan | 1,900 | 29.64 |
| Eastern Mediterranean Region | United Arab Emirates | 1,886 | 29.42 |
| Eastern Mediterranean Region | Saudi Arabia | 1,002 | 15.63 |
| Eastern Mediterranean Region | Tunisia | 386 | 6.02 |
| Eastern Mediterranean Region | Egypt | 249 | 3.88 |
| European Region | United Kingdom | 26,619 | 46.36 |
| European Region | France | 3,641 | 6.34 |
| European Region | Germany | 3,640 | 6.34 |
| European Region | Spain | 3,272 | 5.7 |
| European Region | Netherlands | 2,703 | 4.71 |
| Region of the Americas | United States | 103,974 | 85.34 |
| Region of the Americas | Canada | 12,144 | 9.97 |
| Region of the Americas | Brazil | 1,487 | 1.22 |
| Region of the Americas | Mexico | 1,287 | 1.06 |
| Region of the Americas | Argentina | 609 | 0.5 |
| South-East Asia Region | India | 23,143 | 92.86 |
| South-East Asia Region | Indonesia | 529 | 2.12 |
| South-East Asia Region | Bangladesh | 437 | 1.75 |
| South-East Asia Region | Thailand | 317 | 1.27 |
| South-East Asia Region | Sri Lanka | 250 | 1.0 |
| Western Pacific Region | Australia | 5,567 | 43.77 |
| Western Pacific Region | Singapore | 1,203 | 9.46 |
| Western Pacific Region | Philippines | 1,180 | 9.28 |
| Western Pacific Region | Japan | 1,035 | 8.14 |
| Western Pacific Region | China | 935 | 7.35 |

**Supplemental Table 2. Top national contributors to posts in each WHO region.** Proportions are calculated relative to the total number of posts labelled by GPT in each WHO Region.

**Results**

LLM Validation

| **Domain** | **GPT-4** | **GPT-3.5 Turbo** |
| --- | --- | --- |
| **Overall Sentiment** |  |  |
| Sentiment | Zero-shot: 0.74 (0.67, 0.81) | Few-shot: 0.71 (0.62, 0.79) |
| **Confidence in Health AI** |  |  |
| Safety | Few-shot CoT: 0.79 (0.72, 0.86) | Few-shot CoT: 0.64 (0.56, 0.72) |
| Usefulness | Few-shot CoT: 0.77 (0.69, 0.84) | Few-shot CoT: 0.69 (0.60, 0.76) |
| Trust | Zero-shot CoT: 0.63 (0.56, 0.71) | Zero-shot: 0.61 (0.52, 0.70) |
| Privacy | Few-shot CoT: 0.76 (0.68, 0.82) | Few-shot CoT: 0.66 (0.57, 0.74) |
| Ethics | Few-shot CoT: 0.71 (0.62, 0.78) | Few-shot CoT: 0.63 (0.55, 0.71) |
| Quality | Few-shot CoT: 0.76 (0.67, 0.83) | Few-shot CoT: 0.70 (0.61, 0.78) |

**Supplemental Table 3. Best prompting techniques engine.** Selected best prompts and associated mean wF1-score and 95% CI using bootstrapping n=1,000 resamples.


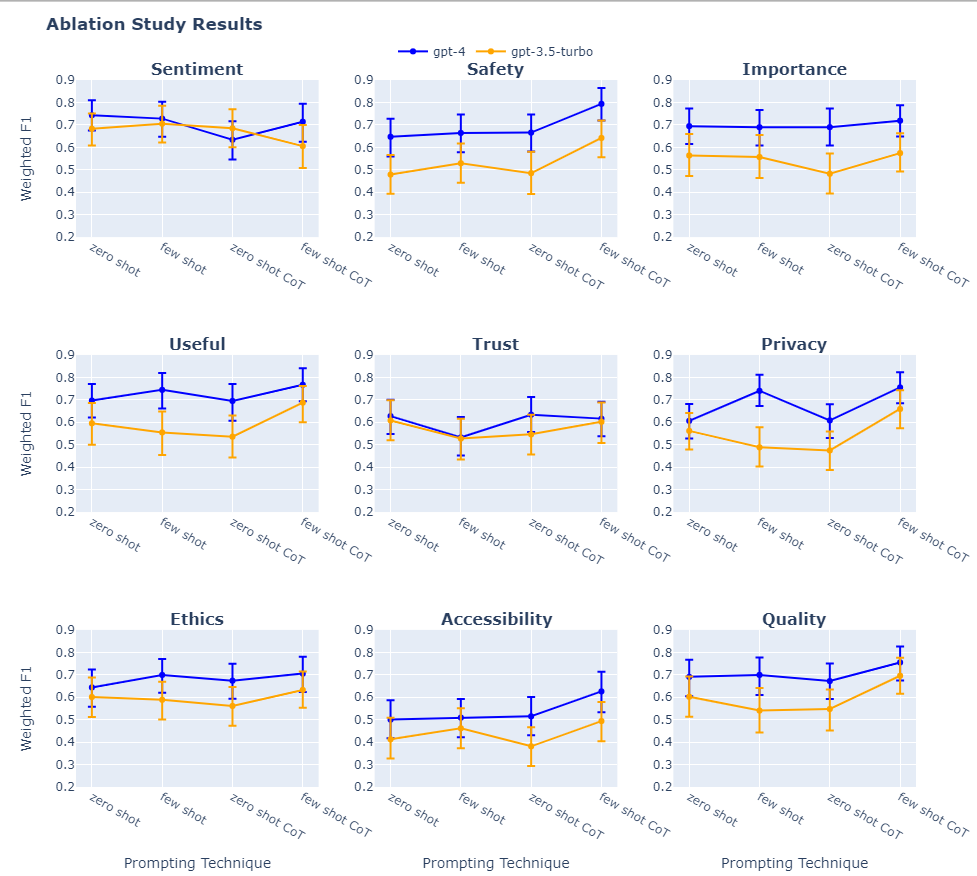


**Supplemental Figure 3. Prompt ablation by health AI confidence domain, including overall sentiment.** While the general trend favors few-shot CoT prompting using both GPT engines, sentiment and trust are the exceptions.

**Sentiment Analysis**

| **Country** | **Negative** | **Neutral** | **Positive** | **Sentiment Score** |
| --- | --- | --- | --- | --- |
| Unknown | 17197 | 37506 | 98440 | 0.53 |
| United States | 9953 | 25856 | 67587 | 0.56 |
| United Kingdom | 2578 | 7117 | 16831 | 0.54 |
| India | 814 | 6504 | 15797 | 0.65 |
| Canada | 1135 | 2691 | 8286 | 0.59 |
| Australia | 547 | 1909 | 3096 | 0.46 |
| Germany | 266 | 751 | 2616 | 0.65 |
| Nigeria | 161 | 925 | 2503 | 0.65 |
| Spain | 129 | 772 | 2359 | 0.68 |
| France | 242 | 1068 | 2313 | 0.57 |
| Netherlands | 191 | 611 | 1898 | 0.63 |
| Switzerland | 114 | 623 | 1850 | 0.67 |
| United Arab Emirates | 34 | 424 | 1424 | 0.74 |
| Pakistan | 77 | 384 | 1422 | 0.71 |
| Kenya | 61 | 396 | 1168 | 0.68 |
| Italy | 118 | 461 | 1163 | 0.60 |
| Belgium | 79 | 324 | 1127 | 0.68 |
| Ireland | 152 | 307 | 991 | 0.58 |
| Singapore | 35 | 204 | 962 | 0.77 |
| South Africa | 146 | 399 | 914 | 0.53 |
| Saudi Arabia | 16 | 204 | 778 | 0.76 |
| Mexico | 98 | 410 | 777 | 0.53 |
| Brazil | 105 | 646 | 729 | 0.42 |
| Philippines | 107 | 383 | 684 | 0.49 |
| Japan | 69 | 326 | 638 | 0.55 |
| Portugal | 56 | 144 | 629 | 0.69 |
| Turkey | 29 | 201 | 579 | 0.68 |
| Tanzania | 65 | 150 | 512 | 0.61 |
| China | 167 | 258 | 499 | 0.36 |
| Ukraine | 22 | 55 | 494 | 0.83 |
| Sweden | 62 | 199 | 417 | 0.52 |
| Uganda | 22 | 115 | 414 | 0.71 |
| Ghana | 32 | 123 | 414 | 0.67 |
| Poland | 55 | 135 | 409 | 0.59 |
| Argentina | 57 | 147 | 404 | 0.57 |
| Malaysia | 39 | 167 | 396 | 0.59 |
| Israel | 27 | 115 | 377 | 0.67 |
| Cameroon | 92 | 157 | 347 | 0.43 |
| Indonesia | 33 | 147 | 346 | 0.60 |
| Colombia | 47 | 119 | 338 | 0.58 |
| Russia | 54 | 134 | 316 | 0.52 |
| New Zealand | 154 | 131 | 295 | 0.24 |
| Jersey | 42 | 552 | 216 | 0.21 |

**Supplemental Table 4. AI sentiment distributions by country.** Counts of posts with positive, neutral, and negative attitudes towards AI over the entire study duration for countries with more than 500 total posts. Sorted by highest number of positive posts.

**Sentiment Analysis**

| **WHO Region** | **Safe** | **Useful** | **Trustworthy** | **Private** | **Ethical** | **Good Quality** |
| --- | --- | --- | --- | --- | --- | --- |
| Overall | 85.4 (85.2–85.6) | 92.4 (92.3–92.5) | 81.3 (81.2–81.5) | 66.7 (66.3–67.0) | 83.3 (83.0–83.5) | 86.5 (86.3–86.6) |
| African Region | 90.7 (89.9–91.6) | 94.8 (94.3–95.3) | 87.3 (86.3–88.3) | 74.2 (72.2–76.2) | 90.6 (89.5–91.6) | 91.2 (90.4–91.9) |
| Eastern Mediterranean Region | 94.0 (93.1–94.8) | 97.1 (96.7–97.6) | 92.3 (91.4–93.3) | 79.6 (76.9–82.3) | 94.6 (93.6–95.8) | 94.5 (93.8–95.3) |
| European Region | 85.7 (85.2–86.1) | 92.8 (92.6–93.0) | 80.7 (80.2–81.2) | 69.5 (68.6–70.4) | 83.5 (82.9–84.2) | 86.1 (85.7–86.5) |
| Region of the Americas | 83.1 (82.7–83.4) | 91.8 (91.7–92.0) | 80.1 (79.8–80.5) | 65.3 (64.7–65.9) | 80.9 (80.5–81.3) | 84.7 (84.4–85.0) |
| South-East Asia Region | 93.1 (92.7–93.6) | 96.4 (96.2–96.7) | 91.8 (91.3–92.3) | 76.0 (74.7–77.4) | 92.4 (91.6–93.1) | 93.0 (92.6–93.5) |
| Western Pacific Region | 82.5 (81.4–83.5) | 91.6 (91.1–92.2) | 78.9 (77.9–80.0) | 67.3 (65.4–69.4) | 81.4 (80.1–82.9) | 84.4 (83.5–85.3) |
| Undisclosed | 85.4 (85.1–85.7) | 91.7 (91.5–91.8) | 80.3 (80.0–80.6) | 64.4 (63.9–65.0) | 82.7 (82.3–83.1) | 86.4 (86.1–86.6) |

**Supplemental Table 5. Health AI Confidence on X.** Sample proportion (95% CI) of posts in support of each health AI concept. Denominator is the total number of posts relevant to each concept (e.g., health AI safety is mentioned) per region.

**Emotion Analysis & Topic Modelling**

| **WHO**  **Region** | **Anger** | **Anticipation** | **Disgust** | **Fear** | **Joy** | **Optimism** | **Pessimism** | **Sadness** | **Trust** |
| --- | --- | --- | --- | --- | --- | --- | --- | --- | --- |
| African Region | 12.87 (12.15–13.6) | 59.48 (58.39–60.51) | 21.8 (20.91–22.73) | 9.07 (8.48–9.73) | 54.68 (53.63–55.78) | 85.72 (84.99–86.47) | 22.79 (21.87–23.73) | 14.47 (13.69–15.22) | 12.6 (11.87–13.31) |
| Eastern Mediterranean Region | 6.32 (5.6–7.03) | 65.1 (63.63–66.58) | 15.19 (14.15–16.13) | 7.42 (6.6–8.23) | 64.11 (62.72–65.46) | 91.63 (90.79–92.4) | 19.51 (18.41–20.65) | 12.03 (11.16–12.95) | 13.3 (12.37–14.29) |
| European Region | 12.94 (12.6–13.26) | 64.42 (63.91–64.91) | 24.84 (24.41–25.29) | 11.77 (11.46–12.1) | 51.64 (51.17–52.14) | 85.11 (84.78–85.43) | 28.16 (27.7–28.6) | 17.24 (16.85–17.63) | 11.13 (10.83–11.47) |
| Region of the Americas | 14.05 (13.82–14.3) | 65.37 (65.06–65.68) | 26.67 (26.37–26.98) | 12.28 (12.07–12.51) | 49.08 (48.75–49.41) | 83.56 (83.3–83.79) | 29.91 (29.59–30.22) | 17.47 (17.21–17.73) | 11.99 (11.77–12.21) |
| South-East Asia Region | 6.27 (5.92–6.67) | 69.45 (68.78–70.11) | 17.41 (16.87–18.0) | 8.51 (8.13–8.9) | 59.73 (59.02–60.45) | 91.8 (91.41–92.2) | 21.91 (21.32–22.53) | 12.06 (11.54–12.55) | 13.98 (13.5–14.5) |
| Undisclosed | 16.08 (15.86–16.3) | 56.53 (56.25–56.81) | 26.64 (26.37–26.9) | 11.24 (11.04–11.43) | 52.16 (51.87–52.45) | 81.59 (81.37–81.83) | 26.56 (26.29–26.83) | 17.32 (17.1–17.53) | 10.19 (10.01–10.37) |
| Western Pacific Region | 18.64 (17.88–19.45) | 61.2 (60.15–62.22) | 31.82 (30.83–32.76) | 16.9 (16.14–17.62) | 50.3 (49.28–51.32) | 80.85 (80.07–81.65) | 32.29 (31.34–33.23) | 22.2 (21.36–23.05) | 10.54 (9.95–11.17) |
| Overall | 14.2 (14.08–14.34) | 61.6 (61.42–61.77) | 25.64 (25.47–25.81) | 11.53 (11.4–11.65) | 51.84 (51.66–52.03) | 83.62 (83.48–83.76) | 27.49 (27.31–27.66) | 17.01 (16.87–17.15) | 11.26 (11.14–11.39) |

**Supplemental Table 6. Emotion analysis by WHO region.** Estimates (95% CI) of proportion of emotions detected in X posts about health AI, using EmoLlama-7b model.

| **Topic name** | **n (% [95% CI])** | **Probable terms** | **Examples** |
| --- | --- | --- | --- |
| Tech industry | 42,399 (15.88% [15.67,15.94]) | business, chatgpt, public, digital, ethical, social, technology, education, system, need | - “Explore the world of AI chatbots across industries! From e-commerce to healthcare, these intelligent tools are reshaping businesses. Discover how they align with industry KPIs and stay updated on the latest innovations. Dive in!” - “As an entrepreneur, I learnt how to use technology to create real impact & pivot my company to be ahead of the curve. Being among the founders of top AI, education & healthcare companies, & understanding the new age findings in AI, AR/VR, robotics & medicine was super insightful.” |
| Algorithms and data | 37,700 (14.05% [13.92,14.19]) | datum, model, patient, medical, care, generative, system, new, clinical, machine learning | - “Our generative AI (really a deconstructed neural network-based expert system model built on established public scientific evidence) enables both patients and clinicians to increase their connection and collaboration leading to better and more deeply personalized healthcare.” - “Excellent article highlighting issues around AI in health care. Notes a number of challenges: "a major issue is the relative scarcity of publicly available data sets in medicine." Reproducibility issues that haunt health-care AI” |
| Human-AI alignment | 37,184 (13.86% [13.72–13.99]) | mental, people, need, human, help, chatgpt, support, work, good, time | - Are you the type of person who wants to stick with the old techniques? If so, It's better if you start learning how to flip the burgers, or wait, even AI will replace these jobs‚ AI is on absolute fire! - “me: for the good of mankind we need to beat up anyone in AI development with hammers Everyone around me: oh so you hate technology huh! People developing Ais” |
| Patient care applications | 33,850 (12.6% [12.48,12.73]) | patient, care, medical, treatment, improve, disease, diagnosis, help, technology, doctor | - “Artificial intelligence is having a MAJOR breakthrough within the healthcare industry right now! It's playing a huge role in the discovery of new drugs, triaging, and fighting specific resistances against drugs.” - “The predictive capabilities of AI tools save invaluable time, allowing #healthcare professionals to focus on what truly matters - their patients. By improving diagnostic processes, #patientsatisfaction, and overall #health outcomes are bound to reach new heights.” |
| Research forums | 33,198 (12.37% [12.25,12.49]) | research, join, global, digital, work, science, innovation, discuss, project, future | - “As we mark #BreastCancerAwarenessMonth, check out this study on AI's transformative role in same-day diagnostics from #AHRQ's Digital Healthcare Research Program 2022 Year in Review.” - “Learn all about federated learning in healthcare projects at AI Meetup next week! Amii's Payam Mousavi leads a panel discussion feat. Ross Mitchell, Ruchika & Neeraj Kumar, three co-authors of a recently published study in @NatureComms” |
| Societal impact | 21,981 (8.19% [8.09,8.29]) | business, chatgpt, public, digital, ethical, social, technology, education, system, need | - “#Italy should follow the example of other countries that are embracing AI and exploring its possibilities. #ChatGPT can be used to improve education, healthcare, and many other areas, and its ban only hinders progress” - “As an optimist and innovator, I wish for the accelerated global impact of AI for better healthcare, better nutrition, better climate adaptation, better education, better resource utilization, better energy sources,....” |
| ChatGPT | 21,131 (7.87% [7.78,7.98]) | chatgpt, question, good, care, new, today, doctor, talk, news, medicine | - “For those of you who have been working on AI in healthcare and always thought AI would not replace physicians, do you still feel the same after LLMs such as Bard and GPT4?” - “Some doctors are using AI chatbots like ChatGPT to communicate with patients â€” even helping doctors deliver bad medical news in a more empathetic way.” |
| Wellness products | 17,084 (6.37% [6.27,6.45]) | startup, app, apple, work, fitness, learn, company, new, build, tech | - “About to launch my new AI passion project for fitness fanatics and those keen on optimising their health Launching tomorrow if things go to plan!” - “10/ Health & Fitness Apps Custom workout & diet plans? AI's on it. Health-conscious folks pay premium for personalization. Generic health advice is passé. Customize with AI, monetize massively.” |
| Task automation | 12,260 (4.57% [4.49,4.65]) | bot, insurance, wellness, healthy, pay, increase, earn, company, telegram, reward | - “Why NO ONE should be signing up for #Medicare #Advantage plans. "Denied by AI: How Medicare Advantage plans use algorithms to cut off care for seniors in need."” - “Frankly I think that companies that use AI workforce over a certain percentage should pay SIGNIFICANTLY more in taxes. Also I want a wealth cap. Also I want income proportional fines. Also I want rent controls. And universal basic income/healthcare/edu. That's all though—” |
| AI takeover | 11,560 (4.31% [4.23,4.38]) | brain, create, people, speech, allow, digital, researcher, continue, advance, thought | - “AI, a label for any Technical skulduggery, is creeping into these spaces from health to banking. On top of dishonest it's dangerously stupid. This will be dangerously out of control very fast.” - “@RishiSunak UK Govt. hasn't yet determined how safe 5G is, & refuses too, it just rolls it out! So, it's doubtful you'll ever bother with health & safety on AI, anymore than @MHRAgovuk did on the mRNA viral gene therapy. It's just public/private money for globalist predators to profit.” |

**Supplemental Table 7. LDA topic model descriptions (n=10).** Named topics, frequency, keywords, and two examples for each topic.
