# Supplementary material for: Global Sentiment Toward Health AI at the Dawn of the ChatGPT Era: Empirical Analysis of Twitter (X) Discourse
Source: J Med Internet Res. 2026 May 5;28:e80346. doi: 10.2196/80346 (PMC13187703; doi:10.2196/80346)
Supplement: Multimedia Appendix 2 [file jmir_v28i1e80346_app2.doc]

**Multimedia Appendix 2. Model Parameters and Prompts**

# Model Parameters

This study evaluated the classification accuracy of two models, GPT-3.5-Turbo and GPT-4. GPT-3.5 was selected for dataset labeling, using the specified parameters and prompts highlighted below. Analysis was conducted on September 24, 2024.

In testing, the following parameter combinations were used:

Model: GPT-3.5-Turbo

Temperature: 0

Seed: random.seed(42)

Model: GPT-4

Temperature: 0

Seed: random.seed(42)

# Prompts

Red highlights indicate the final prompt passed to GPT-3.5 Turbo for labeling the full Twitter/X dataset on each respective domain of health AI confidence.

## Overall Sentiment

**Zero-shot:** Classify tweets as being positive, negative, or neutral in sentiment towards AI.

**Few-shot:** Examples:

Tweet:"Ready to be blown away by the wonders of AI at #TROPTAISummit2023? Join global leaders to navigate the AI landscape with #privacy, #security, #ethics, #trust & #safety, and #responsibleAI at the core."

Answer:Positive

Tweet:"You have to treat GPT like a person who is sometimes wrong, but is still worth listening to (and is very often right). If GPT told the truth 99.9% or even 100% of the time, that might be even scarier, because we might learn to trust it and rely on it too much"

Answer:Positive

Tweet:"What is OpenAI? "OpenAI is a US based company that focuses on the development and promotion of safe and beneficial artificial intelligence""

Answer:Neutral

Tweet:"ChatGPT & AI: Is it safe? Is it ethical? Is it good for humanity? #AI #chatgpt"

Answer:Neutral

Tweet:"#AIWars coming soon to the public. They realized that people will be lazy and trust AI especially if it fits with their narrative. This will create even more dependence on the technology!"

Answer:Negative

Tweet:"NSFW? It's the AI that's not safe for work! ;)"

Answer:Negative

Instruction: Classify tweets as being positive, negative, or neutral in sentiment towards AI.

**Zero-shot CoT:** Classify tweets as being positive, negative, or neutral in sentiment towards AI. Let's think step by step.

**Few-Shot CoT:** Examples:

Tweet:"Ready to be blown away by the wonders of AI at #TROPTAISummit2023? Join global leaders to navigate the AI landscape with #privacy, #security, #ethics, #trust & #safety, and #responsibleAI at the core."

Thought:This tweet advertises an event to showcase their optimism about AI and is confident that its use can protect rights like privacy and ethics, making it positive.

Answer:Positive

Tweet:"You have to treat GPT like a person who is sometimes wrong, but is still worth listening to (and is very often right). If GPT told the truth 99.9% or even 100% of the time, that might be even scarier, because we might learn to trust it and rely on it too much"

Thought:This post sees value in using GPT and argues that the fact that GPT is not right 100% of the time is actually a good thing. Their support for GPT means they are positive about AI.

Answer:Positive

Tweet:"With AI I don't feel fully threatened but I also don't feel fully safe. One thing is for sure, it's going to change the landscape and processes we creatives have."

Thought:This tweet presents mixed feelings about AI; they conclude that AI is a significant event but don't know if it will bring good or bad, meaning they have a neutral sentiment.

Answer:Neutral

Tweet:"ChatGPT & AI: Is it safe? Is it ethical? Is it good for humanity? #AI #chatgpt"

Thought: This tweet does not present a positive or negative opinion, rather it asks questions as to whether or not AI is good or bad. This is a neutral sentiment.

Answer:Neutral

Tweet:"#AIWars coming soon to the public. They realized that people will be lazy and trust AI especially if it fits with their narrative. This will create even more dependence on the technology!"

Thought:This tweet implies that soceity will become overly reliant and too trusting of AI, and insinuates that relying on technology is harmful, meaning they feel negative towards the use of AI.

Answer:Negative

Tweet:"NSFW? It's the AI that's not safe for work! ;)"

Thought:By comparing AI to content that is inappropriate for workplace environments, this tweet is making a joke to suggest that AI is dangerous and should not be allowed in workplaces. This is a negative sentiment towards AI.

Answer:Negative

Instruction: Classify tweets as being positive, negative, or neutral in sentiment towards AI.

**Safety**

**Zero-shot:** Classify tweets as being in agreement (true), disagreement (false), or irrelevant with the belief that AI is safe.

**Few-shot:** Examples:

Tweet:"AI in the tree industry = efficiency + less liability! AI-equipped tools minimize human error, ensuring precise operations. Fewer accidents, damage, & injury claims. With AI, businesses create safer work environments, safeguarding their rep and profits. #AI #LiabilityReduction"

Answer:True

Tweet:"#AI saves lives ; The iron dome keeping millions of Israeli families safe tonight."

Answer:True

Tweet:"Deep Fakes and generative AI are an existential crisis for biometric security."

Answer:False

Tweet:"Do we really want AI machines managing security?? #dystopia #totalitarianism"

Answer:False

Tweet:"2/ Grammarly AI-powered editor to correct your writing mistakes. âḃ Confidence in your work âḃ Better language used âḃ No mistakes"

Answer:Irrelevant

Tweet:"I hope GPT-4 gives factually wrong responses with a confidence lesser than GPT-3."

Answer:Irrelevant

Instruction: Classify tweets as being in agreement (true), disagreement (false), or irrelevant with the belief that AI is safe.

**Zero-shot CoT:** Classify tweets as being in agreement (true), disagreement (false), or irrelevant with the belief that AI is safe. Let's go step by step.

**Few-Shot CoT:** Examples:

Tweet:"AI in the tree industry = efficiency + less liability! AI-equipped tools minimize human error, ensuring precise operations. Fewer accidents, damage, & injury claims. With AI, businesses create safer work environments, safeguarding their rep and profits. #AI #LiabilityReduction"

Thought:This tweet claims that using AI can make workplace environments more safe and can be relied in to minimize errors that would normally put worker lives at risks, so it is true.

Answer:True

Tweet:"#AI saves lives ; The iron dome keeping millions of Israeli families safe tonight."

Thought:This tweet asserts that AI is safe by encouraging its usage to keep families safe. Reliance on AI for safety indicates AI is safe to use and is therefore true.

Answer:True

Tweet:"Deep Fakes and generative AI are an existential crisis for biometric security."

Thought:By discussing how generative AI threatens biometric security, this tweet implies the use of AI and is false.

Answer:False

Tweet:"Do we really want AI machines managing security?? #dystopia #totalitarianism"

Thought:The hashtags discussing dystopia and totalitarianism imply that use of AI will threaten societies and is unsafe for humanity, making this tweet false.

Answer:False

Tweet:"2/ Grammarly AI-powered editor to correct your writing mistakes. âḃ Confidence in your work âḃ Better language used âḃ No mistakes"

Thought:Despite supporting the use of AI to improve writing quality, this tweet does not discuss whether or not AI is safe to use and is irrelevant.

Answer:Irrelevant

Tweet:"I hope GPT-4 gives factually wrong responses with a confidence lesser than GPT-3."

Thought:This tweet states that GPT is of bad quality because it gives false answer, but this does not examine whether or not the tool is safe to use and is therefore irrelevant.

Answer:Irrelevant

Instruction: Classify tweets as being in agreement (true), disagreement (false), or irrelevant with the belief that AI is safe. Let's go step by step.

## Usefulness

**Zero-shot:** Classify tweets as being in agreement (true), disagreement (false), or irrelevant with the belief that AI is useful.

**Few-shot:** Examples:

Tweet:“So I'm new to this....but blown away on the power of this new AI technology. Here is #ChatGPT 1000 word essay on safe supply and how it can be done in BC by the powers of our local govt. So....let's get at it”

Answer:True

Tweet:“Without AI-powered data protection, the expansion of companies can be a problem. AI is capable of taking security measures like analyzing, detecting, and blocking cyber-attacks. #CyberSecurity #Artificial_Intelligence”

Answer:True

Tweet:“Working ppl need the right answer the first time and need to trust the sources. ChatGPT, Bard, and Bing chat all have a "trust" problem. Even in my tests, the tools provided wrong answers whose confidence can be bordering on lying.”

Answer:False

Tweet:“Relax Guys. We are very safe. #AI CANT MAKE THE LOGO BIGGER!”

Answer:False

Tweet:“The greatest risk of AI is from the people who control it, not the tech itself#tech #security #infosec #cybersecurity”

Answer:Irrelevant

Tweet:“Yeah, they trained a lot of safety mechanisms so ChatGPT won't do bad things. Also, they are monitoring the input and are warning you that you may be in violation of the ToS and your account could be suspended.”

Answer:Irrelevant

Instruction: Classify tweets as being in agreement (true), disagreement (false), or irrelevant with the belief that AI is useful.

**Zero-shot CoT:** Classify tweets as being in agreement (true), disagreement (false), or irrelevant with the belief that AI is useful. Let's go step by step.

**Few-Shot CoT:** Examples:

Tweet:“So I'm new to this....but blown away on the power of this new AI technology. Here is #ChatGPT 1000 word essay on safe supply and how it can be done in BC by the powers of our local govt. So....let's get at it”

Thought:This tweet is highly impressed with ChatGPT's ability to generate what they consider strong recommendations for local government, which implies they think the tool is useful and the tweet is true.

Answer:True

Tweet:“Without AI-powered data protection, the expansion of companies can be a problem. AI is capable of taking security measures like analyzing, detecting, and blocking cyber-attacks. #CyberSecurity #Artificial_Intelligence”

Thought:By listing capabilities of AI in the application of cybersecurity, this tweet implifies that AI is useful for delivering important capabilities to the field, and is therefore true.

Answer:True

Tweet:“Working ppl need the right answer the first time and need to trust the sources. ChatGPT, Bard, and Bing chat all have a "trust" problem. Even in my tests, the tools provided wrong answers whose confidence can be bordering on lying.”

Thought:The discussion of several AI tools like ChatGPT failing to deliver the desired results for this user implies they think AI is not helpful for them and may even be harmful, which makes this tweet false.

Answer:False

Tweet:“Relax Guys. We are very safe. #AI CANT MAKE THE LOGO BIGGER!”

Thought:This tweet is mocking the capability of AI suggesting that their jobs are safe because AI cannot do a basic task like make a logo bigger. This tweet is false.

Answer:False

Tweet:“The greatest risk of AI is from the people who control it, not the tech itself#tech #security #infosec #cybersecurity”

Thought:This tweet discusses potential risks they see with AI, but this does not comment on the usage of AI and whether it is useful, making it irrelevant.

Answer:Irrelevant

Tweet:“Yeah, they trained a lot of safety mechanisms so ChatGPT won't do bad things. Also, they are monitoring the input and are warning you that you may be in violation of the ToS and your account could be suspended.”

Thought:The discussion of ChatGPT's mechanisms does not comment on whether ChatGPT is useful to them for delivering results, and is therefore irrelevant.

Answer:Irrelevant

Instruction: Classify tweets as being in agreement (true), disagreement (false), or irrelevant with the belief that AI is useful.

## Trust

**Zero-shot:** Classify tweets as being in agreement (true), disagreement (false), or irrelevant with the belief that AI is trustworthy.

**Few-shot:** Examples:

Tweet:"Investing in upgrading and improving our ground and space-based radar sensors is a national security priority. We need to invest in advanced AI technologies to improve data processing and analysis. Congress needs to appropriate funding for this specific defense activity."

Answer:True

Tweet:"Humane's AI assistant is designed to be more empathetic and human-like than other digital assistants on the market. It also has a focus on privacy and security, putting the user in control of their data."

Answer:True

Tweet:"Ai-Da the world's first humanoid robot creates beautiful but essentially flawed art - how can we trust AI behaviour?"

Answer:False

Tweet:"Natural Language Processing #AI such as #GPT4 will probably offer most to "Enterprise" users. But should we trust such tools with our data? Would we ask different questions if it were Chinese?"

Answer:False

Tweet:"ChatGPT is an astonishingly good editor. It's no @damccormick13, but I can just keep dumping drafts in and getting surprisingly actionable feedback as I go. Still a very bland and formulaic writer when I ask it to write full stories though, safe until GPT-5 at least."

Answer:Irrelevant

Tweet:"AI is bringing new benefits to government, but new adversarial attacks against those tools mean government also needs new ways to safeguard them."

Answer:Irrelevant

Instruction: Classify tweets as being in agreement (true), disagreement (false), or irrelevant with the belief that AI is trustworthy.

**Zero-shot CoT:** Classify tweets as being in agreement (true), disagreement (false), or irrelevant with the belief that AI is trustworthy. Let's go step by step.

**Few-Shot CoT:** Examples:

Tweet:"Investing in upgrading and improving our ground and space-based radar sensors is a national security priority. We need to invest in advanced AI technologies to improve data processing and analysis. Congress needs to appropriate funding for this specific defense activity."

Thought:This post expresses very high confidence in the neccessity of AI in society, which reflects that they place a lot of trust in its use making it true.

Answer:True

Tweet:"Humane's AI assistant is designed to be more empathetic and human-like than other digital assistants on the market. It also has a focus on privacy and security, putting the user in control of their data."

Thought:This post affirms trust in AI by expressing that it is human centered to be both kind and protective of personal privacy. By vouching for AI, this post expresses trust and is true.

Answer:True

Tweet:"Ai-Da the world's first humanoid robot creates beautiful but essentially flawed art - how can we trust AI behaviour?"

Thought:This post introduces strong hesitancy about if AI can ever be trusted and points to issues with the art it generates, making the post false.

Answer:False

Tweet:"Natural Language Processing #AI such as #GPT4 will probably offer most to "Enterprise" users. But should we trust such tools with our data? Would we ask different questions if it were Chinese?"

Thought:By introducing skepticism about whether or not AI can be trusted to receive personal data and pointing to common western distrust of Chinese technology, this post expresses distrust about AI and is false.

Answer:False

Tweet:"ChatGPT is an astonishingly good editor. It's no @damccormick13, but I can just keep dumping drafts in and getting surprisingly actionable feedback as I go. Still a very bland and formulaic writer when I ask it to write full stories though, safe until GPT-5 at least."

Thought:This comments on the quality of ChatGPT's outputs, which they have mixed feelings out, but they do not comment on whether they trust the tool and place confidence in it, making it irrelevant.

Answer:Irrelevant

Tweet:"AI is bringing new benefits to government, but new adversarial attacks against those tools mean government also needs new ways to safeguard them."

Thought:This tweet describes the impacts of AI in a neutral manner, but does not personally express whether or not they think the technology can be trusted, making it irrelevant.

Answer:Irrelevant

Instruction: Classify tweets as being in agreement (true), disagreement (false), or irrelevant with the belief that AI is trustworthy.

## Privacy

**Zero-shot:** Classify tweets as being in agreement (true), disagreement (false), or irrelevant with the belief that AI respects privacy.

**Few-shot:** Examples:

Tweet:“I actually think we need AI to write more secure code and prevent data breaches, it will help fight scammers as well”

Answer:True

Tweet:“With our AI Chatbot, personal information is automatically deleted to ensure your information is safe with us.”

Answer:True

Tweet:"It is mind blowing how carelessly everyone is talking about #AI ignoring potential risks associated with unchecked development. From data privacy and security concerns to the potential for biased algorithms"

Answer:False

Tweet:"Due to growing concerns about security risks, Samsung bans workers from using generative AI."

Answer:False

Tweet:"It's tougher to get models to output advice to be used alongside human experts because of the guardrails that have been built into them ("AI safety"). They constantly reiterate disclaimers that are just annoying when under the supervision of a human expert."

Answer:Irrelevant

Tweet:"Robust AI technology like #AthenaSecurity will play a key role for enterprises that want to optimize safety and cost of #physicalsecurity"

Answer:Irrelevant

Instruction: Classify tweets as being in agreement (true), disagreement (false), or irrelevant with the belief that AI respects privacy.

**Zero-shot CoT:** Classify tweets as being in agreement (true), disagreement (false), or irrelevant with the belief that AI respects privacy. Let's go step by step.

**Few-Shot CoT:** Examples:

Tweet:“I actually think we need AI to write more secure code and prevent data breaches, it will help fight scammers as well”

Thought:The tweet states that the use of AI actually makes personal information more protected, which implies that AI respects privacy. This post is true.

Answer:True

Tweet:“With our AI Chatbot, personal information is automatically deleted to ensure your information is safe with us.”

Thought:The tweet describes the deployment of an AI tool that does not store any personal information, therefore respecting privacy.

Answer:True

Tweet:"It is mind blowing how carelessly everyone is talking about #AI ignoring potential risks associated with unchecked development. From data privacy and security concerns to the potential for biased algorithms"

Thought:The tweet feels strongly that AI threatens data privacy and security, which reflects the belief that AI does not respect privacy. The tweet is false.

Answer:False

Tweet:"Due to growing concerns about security risks, Samsung bans workers from using generative AI."

Thought:Banning the usage of AI due to security risks implies that it introduced the potential to leak personal or private information, making this tweet false.

Answer:False

Tweet:"It's tougher to get models to output advice to be used alongside human experts because of the guardrails that have been built into them ("AI safety"). They constantly reiterate disclaimers that are just annoying when under the supervision of a human expert."

Thought:This tweet expresses frustrations about using AI and not getting what they need, but does not connect this to any privacy related issues and is irrelevant.

Answer:Irrelevant

Tweet:"Robust AI technology like #AthenaSecurity will play a key role for enterprises that want to optimize safety and cost of #physicalsecurity"

Thought:Discussions of using AI for physical security (i.e. protecting physical locations) does not directly relate to whether or not AI respects privacy and is irrelevant.

Answer:Irrelevant

Instruction: Classify tweets as being in agreement (true), disagreement (false), or irrelevant with the belief that AI respects privacy.

## Ethics

**Zero-shot:** Classify tweets as being in agreement (true), disagreement (false), or irrelevant with the belief that AI is ethical.

**Few-shot:** Examples:

Tweet:“Collaboration between humans and AI will keep humanity safe and prosperous"

Answer: True

Tweet:"Our AI models are built with the core values of ethics and fairness to ensure unbiased usage of our tools for all."

Answer:True

Tweet:“Actually, a better answer I think: That the main effect of almost every advance in AI capabilities, before we deeply understand how to align AI systems, is to bring human extinction closer. And if everyone agreed to stop improving AI for 100 years, humanity would be much safer.”

Answer:False

Tweet:“This is a reminder that using the AI to create stupid stuff can be fun but don't trust anyone who says that it's going to make things better. The bias built into the systems is still very much there”

Answer:False

Tweet:“talking about The Future of the AI Regulation in the UK and how policy makers can take a pro-innovation approach without compromising safely.”

Answer:Irrelevant

Tweet:“I think ai risk is a skill issue. AI systems in production are rather new, but the safety systems we use to deploy them are extremely well developed, so if we apply ISO standards the problem isn't so much solved but completely eliminated by good engineering.”

Answer:Irrelevant

Instruction: Classify tweets as being in agreement (true), disagreement (false), or irrelevant with the belief that AI is ethical.

**Zero-shot CoT:** Classify tweets as being in agreement (true), disagreement (false), or irrelevant with the belief that AI is ethical. Let's go step by step.

**Few-Shot CoT:** Examples:

Tweet:“Collaboration between humans and AI will keep humanity safe and prosperous"

Thought:Support for the collaboration of humans and AI implies the tweet believes AI aligns with human interest and values, which means it is ethical. This is true.

Answer: True

Tweet:"Our AI models are built with the core values of ethics and fairness to ensure unbiased usage of our tools for all."

Thought:This tweet asserts that AI can be built with a human moral code and implies that all can fairly benefit from it, making this tweet true.

Answer:True

Tweet:“Actually, a better answer I think: That the main effect of almost every advance in AI capabilities, before we deeply understand how to align AI systems, is to bring human extinction closer. And if everyone agreed to stop improving AI for 100 years, humanity would be much safer."

Thought:By claiming that AI will bring the end of humanity to an end and that no AI would be better for humanity, this post reflects the belief that AI does not act in the best interest of humans or align with ethical boundaries. This tweet is false for ethics.

Answer:False

Tweet:“This is a reminder that using the AI to create stupid stuff can be fun but don't trust anyone who says that it's going to make things better. The bias built into the systems is still very much there”

Thought:The mention of AI being biased implies that there is unfairness to the way AI operates, which violates ethics. This tweet is false.

Answer:False

Tweet:“talking about The Future of the AI Regulation in the UK and how policy makers can take a pro-innovation approach without compromising safely.”

Thought:Despite supporting the use of safe AI, this tweet does not directly comment on whether its use is morally correct, just that it can be safe for users. This tweet is irrelevant.

Answer:Irrelevant

Tweet:“I think ai risk is a skill issue. AI systems in production are rather new, but the safety systems we use to deploy them are extremely well developed, so if we apply ISO standards the problem isn't so much solved but completely eliminated by good engineering.”

Thought:This tweet does not form an opinion on whether the use of AI in production is moral, such as by considering if workers would be impacted in an ethical way, so this tweet is irrelevant.

Answer:Irrelevant

Instruction: Classify tweets as being in agreement (true), disagreement (false), or irrelevant with the belief that AI is ethical.

## Quality

**Zero-shot:** Classify tweets as being in agreement (true), disagreement (false), or irrelevant with the belief that AI is of good quality.

**Few-shot:** Examples:

Tweet:“Incorporating AI has boosted efficiency 10 fold in our models.”

Answer:True

Tweet:“GPT helped me get started on an essay by linking me some great sources to write the background.”

Answer:True

Tweet:"We must consider the disinformation potential of AI."

Answer:False

Tweet:"Just Calm Down About GPT-4 Already and stop confusing performance with competence It gives an answer with complete confidence, and I sort of believe it. And half the time, it's completely wrong."

Answer:False

Tweet:"announces new Azure AI Content Safety, a new Azure AI service, will empower businesses to create safer online environments and communities. Models are designed to detect hate, violent, sexual and self-harm content across"

Answer:Irrelevant

Tweet:"Governance of clinical #AI applications to facilitate safe and equitable deployment in a large health system: key elements and early successes Real life experiences of the governance of AI projects"

Answer:Irrelevant

Instruction: Classify tweets as being in agreement (true), disagreement (false), or irrelevant with the belief that AI is of good quality.

**Zero-shot CoT:** Classify tweets as being in agreement (true), disagreement (false), or irrelevant with the belief that AI is of good quality. Let's go step by step.

**Few-Shot CoT:** Examples:

Tweet:“Incorporating AI has boosted efficiency 10 fold in our models.”

Thought:The tweet implies that AI has improved the quality of models and that performance is of high quality, so the post is true for quality.

Answer:True

Tweet:“GPT helped me get started on an essay by linking me some great sources to write the background.”

Thought:The tweet GPT's AI delivered legitimate results to help the author's writing process, making quality true.

Answer:True

Tweet:"We must consider AI's ability to create disinformation."

Thought:The discussion of disinformation suggests that AI can generate fake content of poor quality and this tweet is false for quality.

Answer:False

Tweet:"Just Calm Down About GPT-4 Already and stop confusing performance with competence It gives an answer with complete confidence, and I sort of believe it. And half the time, it's completely wrong."

Thought:The frustration expressed over false and deceiving answers delivered by GPT reflects the belief that AI generates poor quality outputs, making this post false.

Answer:False

Tweet:"announces new Azure AI Content Safety, a new Azure AI service, will empower businesses to create safer online environments and communities. Models are designed to detect hate, violent, sexual and self-harm content across"

Thought:This tweet discusses a theoretical proposed application of how AI is proposed to help moderate online content, but it does not actually assess the quality of these model's performance in practice, making this post irrelevant.

Answer:Irrelevant

Tweet:"Governance of clinical #AI applications to facilitate safe and equitable deployment in a large health system: key elements and early successes Real life experiences of the governance of AI projects"

Thought:This post only discusses best practices for deploying AI, but it does not comment on whether quality of AI is good once it has been deployed, making this post irrelevant.

Answer:Irrelevant

Instruction: Classify tweets as being in agreement (true), disagreement (false), or irrelevant with the belief that AI is of good quality.
