# Supplementary material for: Global Sentiment Toward Health AI at the Dawn of the ChatGPT Era: Empirical Analysis of Twitter (X) Discourse
Source: J Med Internet Res. 2026 May 5;28:e80346. doi: 10.2196/80346 (PMC13187703; doi:10.2196/80346)
Supplement: Multimedia Appendix 3 [file jmir_v28i1e80346_app3.doc]

**AI Usage Consideration Checklist**


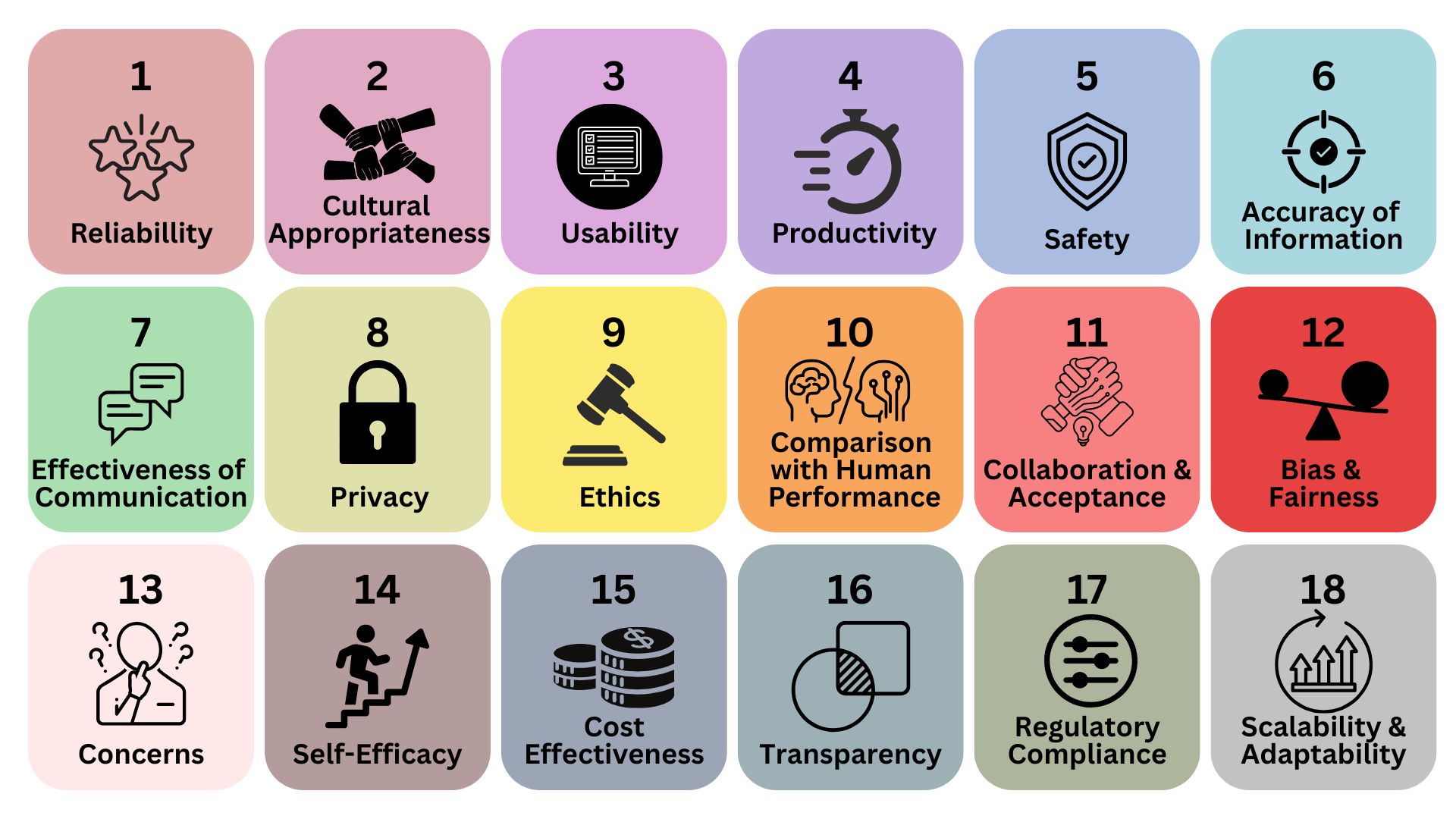


**Supplemental Appendix Figure 3.** Visual Framework of the AI Usage Consideration Checklist

| **Item** | **Domain** | **Questions** |
| --- | --- | --- |
| 1 | Reliability | • Does the AI consistently perform its designated tasks effectively?  • Have testing and validation processes confirmed its reliability under various conditions? |
| 2 | Cultural Appropriateness | • Does the AI adapt appropriately to cultural, linguistic, and societal norms?  • Are its outputs and interactions respectful and relevant across diverse cultural contexts? |
| 3 | Usability | • Is the AI intuitive and easy to use for all user groups?  • Are onboarding resources and technical support readily available? |
| 4 | Productivity | • Does the AI demonstrably enhance efficiency or productivity?  • Are the gains in productivity sustainable and scalable? |
| 5 | Safety | • Does the AI operate safely in its intended environment?  • Are there protocols for addressing potential hazards or malfunctions? |
| 6 | Accuracy of Information | • Is the AI’s output accurate, relevant, and contextually appropriate?  • Are mechanisms in place to identify and correct inaccuracies? |
| 7 | Effectiveness of Communication | • Does the AI communicate in a clear, concise, and audience-appropriate manner?  • Are communication errors tracked and addressed? |
| 8 | Privacy | • Does the AI comply with applicable privacy laws and regulations?  • Are there robust systems to safeguard personal data from unauthorized access? |
| 9 | Ethics | • Does the AI align with organizational and societal ethical standards?  • Are potential ethical dilemmas identified and proactively managed? |
| 10 | Comparison with Human Performance | • How does the AI’s performance compare to humans in similar roles or tasks?  • Are the AI's strengths and limitations well-documented? |
| 11 | Collaboration and Acceptance | • Are stakeholders and users open to working alongside the AI?  • Is there a clear division of responsibilities between AI and humans? |
| 12 | Bias and Fairness | • Are the AI’s decisions free from bias and unfair discrimination?  • Are there regular audits to detect and address bias? |
| 13 | Concerns | • Are there unresolved concerns regarding the AI’s impact, transparency, or usability?  • Is there a system for documenting and addressing these concerns? |
| 14 | Self-Efficacy | • Are users confident in their ability to use the AI effectively?  • Is adequate training provided on the AI’s capabilities and risks? |
| 15 | Cost-Effectiveness | • Does the AI provide value relative to its cost of implementation and maintenance?  • Are the long-term costs, including updates and potential downtime, accounted for? |
| 16 | Transparency | • Are the AI’s decision-making processes and algorithms explainable and transparent?  • Can users and stakeholders understand how outputs are generated? |
| 17 | Regulatory Compliance | • Does the AI comply with all relevant industry standards and legal requirements?  • Are there mechanisms to ensure ongoing compliance as regulations evolve? |
| 18 | Scalability and Adaptability | • Can the AI scale to meet increasing demands or adapt to new challenges?  • Are updates and improvements feasible without significant disruption? |

**Supplemental Table 8.** AI Usage Consideration Checklist Items
